# A mass spectrometry imaging approach on spatiotemporal distribution of multiple alkaloids in *Gelsemium elegans*

**DOI:** 10.3389/fpls.2022.1051756

**Published:** 2022-11-18

**Authors:** Zi-Han Wu, Ruo-Zhong Wang, Zhi-Liang Sun, Yi Su, Lang-Tao Xiao

**Affiliations:** ^1^ College of Bioscience and Biotechnology, Hunan Agricultural University, Changsha, China; ^2^ College of Veterinary Medicine, Hunan Agricultural University, Changsha, China

**Keywords:** mass spectrometry imaging, alkaloid, *Gelsemium elegans*, spatio-temporal distribution, DESI-MSI

## Abstract

*Gelsemium elegans* contains multiple alkaloids with pharmacological effects, thus researchers focus on the identification and application of alkaloids extracted from *G. elegans*. Regretfully, the spatiotemporal distribution of alkaloids in *G. elegans* is still unclear. In this study, the desorption electrospray ionization mass spectrometry imaging (DESI-MSI) was applied to simultaneously analyze the distribution of pharmacologically important alkaloids in different organ/tissue sections of *G. elegans* at different growth stages. Finally, 23 alkaloids were visualized in roots, stems and leaves at seedling stage and 19 alkaloids were observed at mature stage. In mature *G. elegans*, 16 alkaloids were distributed in vascular bundle region of mature roots, 15 alkaloids were mainly located in the pith region of mature stems and 2 alkaloids were enriched in epidermis region of mature stems. A total of 16 alkaloids were detected in leaf veins of mature leaves and 17 alkaloids were detected in shoots. Interestingly, diffusion and transfer of multiple alkaloids in tissues have been observed along with the development and maturation. This study comprehensively characterized the spatial metabolomics of *G. elegans* alkaloids, and the spatiotemporal distribution of alkaloid synthesis. In addition, the results also have reference value for the development and application of *Gelsemium elegans* and other medicinal plants.

## Introduction

Mass spectrometry imaging (MSI) emerges as an effective biological imaging technique within the last 20 years because of its versatility, high sensitivity, and label-free advantage (Dilillo et al., 2017). Being an important technique to understand the locations of molecular entities inside biochemical and biological systems (Jeckel et al., 2020), it can also capture snapshots for the spatial distribution of metabolites in complex samples, providing an additional dimension of information for metabolic researches. Sample preparation for MSI does not require whole-tissue homogenate, furthermore, within-tissue spatial distribution profiles of metabolites in plants can be acquired through MSI (Bong et al., 2016). Recently, MSI has been employed to visualize certain target molecules in various plant organs, such as *Hypericum perfortum* roots (Tocci et al., 2018), *Ginkgo biloba* leaves (Li et al., 2018), *Vitex agnus-castus* fruits/leaves (Heskes et al., 2018) and strawberry fruits (Enomoto et al., 2018). Meanwhile, it has been used to visualize the spatial distribution of some important medicinal compositions in medicinal plants *Catharanthus roseusm* (Dutkiewicz et al., 2021) and *Camellia sinensis* (Liao et al., 2019).

Alkaloids are naturally occurring cyclic nitrogen containing compounds with low molecular-weight and alkali-like properties, mostly derived from amino acids (Cordell, 2013). As secondary metabolites found in approximately 20% of plant species, alkaloids are reported to play defensive roles against herbivores and pathogens (Lee et al., 2013). In addition, the applications of alkaloids also have pharmacological, veterinary and medical importance (Ziegler and Facchini, 2008). Owing to their diverse effects, many of the approximately 12,000 known alkaloids have been exploited as pharmaceuticals, stimulants, narcotics, and poisons (Facchini, 2001). For example, vinblastine can be used for the treatment of cancer (Souza et al., 2017), and ajmaline can be used for antiarrythmic heart disorders (Ozkartal et al., 2019).

*Gelsemium elegans* (*G. elegans*) is a genus of flowering plants in the *Gelsemicaeae* family being mainly distributed in North America and Southeast Asia (Jin et al., 2014). Previous studies showed that *G. elagans* has a variety of pharmacological effects including anti-tumor, anti-inflammation, skin ulcers relief, immune function, and analgesia (Liu et al., 2011; Ye et al., 2019). In fact, the basic pharmaceutically active compounds in *G. elagans* are alkaloids. According to the chemical structures, *G. elagans* alkaloids are divided into 6 types of gelsedine-, gelsemine-, humantenine-, koumine-, sarpagine- and the yohimbane-type (Jin et al., 2014). Structures of multiple alkaloids in *G. elagans* were characterized by high-performance liquid chromatography coupled with quadruopole time-of-flight mass spectrometry (LC-QTOF/MS) (Liu et al., 2017a) and nuclear magnetic resonance (NMR) (Wang et al., 2017). Meanwhile, the relative quantification is usually carried out through liquid chromatography coupled with mass spectrometry (LC-MS) (Liu et al., 2017b). Furthermore, an offline preparative three-dimensional HPLC (3D-HPLC) method was developed to perform the systematic purification of 24 indole alkaloids in *G. elegans* (Liu et al., 2021). Based on the two-dimension LC-UV-MS plus online heart-cutting method, a total of 256 alkaloids were grouped and tentatively identified in *G. elegans* (Liu et al., 2022).

As one of MSI-based technologies, DESI has firstly been applied in identification and visualization of plant target compounds, such as flavones and alkaloids. Recently, high-performance thin layer chromatography coupled with desorption electrospray ionization multistage mass spectrometry (HPTLC-DESI-MS^n^) analysis was used to identify 13 aporphine and 4 benzylisoquinoline-type alkaloids in *Ocotea spixiana* (Conceicao et al., 2020). The seven *Uncaria* alkaloids were quantitatively imaged in rat brains by using DESI-MSI (Gao et al., 2022). Visualizing the distribution of a metabolite is an important approach to explore its translocation and the possible pathways of biosynthesis (Ifa et al., 2011; Kumara et al., 2019). High-throughput determination of alkaloids is necessary for the research of plant spatial metabolomics. Although DESI-MSI has been applied in the quantification of plant alkaloids, the application in high-throughput determination is rare. In our previous study, we employed DESI-MSI to visualize the distributions of several alkaloids in *G. elegans* (Wu et al., 2022b). Regretfully, the spatiotemporal distribution of more alkaloids in *G. elagans* is still unclear. For better exaction and application, it is essential to determine the detailed tissue localization and putative mobility of alkaloids in *G. elagans*. In this study, we simultaneously analyzed the spatiotemporal distributions of multiple alkaloids in organs/tissues of *G. elegans* at different growth stages by using the desorption electrospray ionization mass spectrometry imaging (DESI-MSI). The results also showed reference value for the development and application of *G. elegans* and other medicinal plants.

## Materials and methods

### Reagents and chemicals

Methanol was obtained from Merck Chemicals Co., Ltd. (Darmstadt, Germany). Saccharose was purchased from the Sinopharm Chemical Reagent Co., Ltd. (Shanghai, China). Glass slides were obtained from Wuhan Servicebio Technology Co., Ltd. (Wuhan, China). Optimum Cutting Polymer (O.C.T.) was purchased from Sakura (USA). Ultrapure water (resistivity ≥ 18.25 MΩ/cm) obtained from WaterPro water system (ULUPURE, China). All the reagents and chemicals employed in the experiments were of superior analytical grade (least 98% purity).

### Plant culture conditions

The seeds of *G. elegans* were collected from Longyan (N24°43′12″, E116°43′48″), Fujian Province, China. The seeds were washed with sterile distilled water, and vernalization at 4 °C was carried out for 48 h. The seeds were germinated and transplanted into sand pots. The potted seedlings were grown in greenhouse with a photoperiod of 16/8 h light/darkness, 70% humidity, and a temperature of 24/20 °C day/night. For DESI-MSI analysis, the seedlings (30 d, 60 d and 90 d after planting) and the mature plants (180 d after planting) of *G. elegans* were collected.

### Alkaloid analysis by LC-MS/MS

The sample preparation method and analytical method for LC-MS/MS was analyzed as previously reported (Wu et al., 2022b). Fresh tissues of *G. elegans* were ground with liquid nitrogen and 100 mg aliquot of the homogenate was immediately collected in microcentrifuge tube. Each tissue has three replicates. The homogenate was extracted twice by ultrasonic bath (Shumei KQ-250DE, Jiangsu, China) in 2.5 mL of 80% ethanol (1:25) for 0.5 h at 60 °C. The extraction solution was combined for filtration, and 1 mL of the filtered solution was evaporated by nitrogen. The sample was prepared by dissolving it in 1 mL of acetonitrile-ammonium acetate (1:4, volume percent) and filtering through a 0.22-µm membrane filter, 10 µL of which was used to LC-MS/MS analysis.

The UPLC-MS/MS was equipped with an Agilent 1290 series liquid chromatography (Agilent Technologies, USA) and Agilent 6460 triple quadruple mass spectrometer (Agilent Technologies, USA). The sample extraction solutions were separated on a Waters C18 column (3.5 μm, 4.6 μm×150 mm, Waters, USA). The separation conditions were as follows: the column oven temperature was kept at 40 °C, and the flow rate of the mobile phase was 0.3 mL/min. Auto MS/MS analysis was performed in both scan mode and multiple reaction monitoring (MRM) mode. Nitrogen gas was used as the drying and collision gas. The ionization source conditions were set as follows: flow rate of the nebulizer gas, 12 L/min; source temperature of the mass spectrometer, 350 °C; nebulizer pressure, 40 psi; capillary voltage, 3.50 kV; scan range, *m/z* 50-1000. The data acquisition was used by Agilent MassHunter workstation software (version B.07.00).

### Plant sample preparation for DESI-MSI

Frozen sections of *G. elegans* organ/tissue were made for DESI-MSI. Fresh organ/tissue samples (root, stem, leaf and shoot) were immediately wrapped in aluminum foils and were frozen in liquid nitrogen. Three replicates of organ/tissue samples were made into frozen sections. The frozen samples were then transferred to a freezer (Haier DW-86L388J, Qingdao, China) for storage at -80 ℃. The frozen samples were put up on the specimen chuck, and O.C.T. embedding agent was dropped around the organ/tissue. The specimen chuck with sample was put on the quick-freezing table of the frozen section machine (Thermo CRYOSTAR NX50, San Jose, CA, USA). When O.C.T. became white and hard, the samples were sectioned with the slice thickness 8-10 µm. The roots, stems and leaves were neatly sliced along their cross sections. The shoots were longitudinally sliced. The organ/tissue sections were adhered to a glass slide and was analyzed by DESI-MSI.

### DESI-MSI instrumentation

DESI-QToF-MS (Waters Xevo G2-XS, Milford, MA, USA) was employed for MSI analysis. Methanol: water=98:2 (v/v) was used as the solvent and was sprayed at an angle of 60° to surface. The flow rate was 2 μL/min. Nebulizing gas (nitrogen) pressure was 5 bar. Distance between the emitter and the mass spectrometer inlet was kept at 3 mm, the emitter was positioned 2 mm above the tissue surface, and the mass spectrometer inlet was 0.5 mm to the tissue surface. Imaging area was chosen according to the sample dimensions, the spatial resolution used was 50 μm × 50 μm, and the spatial scanning rate was 100 μm/s. Imaging acquisition interval varied between 6.0 and 6.5 hours in 1 cm × 1 cm area of tissue sample. Data were acquired in positive ion mode with a spray voltage of 4.5 kV. Signals were recorded from *m/z* 50 to 1200 and the ion source temperature was 150°C. Each experiment has three replicates. Each frozen section was scanned three times by DESI-MSI.

### Compounds imaging and data processing

The acquisition setup, processing, and visualization of data were performed using High Definition Imaging (HDI) 1.5 (Waters Corporation, Manchester, UK) software. Data were acquired and mined using MassLynx software version 4.1 (Waters Corporation, Manchester, UK). To identify the metabolites, each interested peak was scanned for the details of MS^2^ spectrum. The ion intensity values of the organs/tissues were obtained from the mass spectrum exported from the HDI software with centroid data. The image brightness intensities were calculated according to the ion intensity of samples and were plotted as the color scale. The relative quantification of alkaloids was performed according to the image brightness intensities captured by the DESI-MSI.

## Results

### Alkaloids identified in *G. elegans*

**Figure 1:**
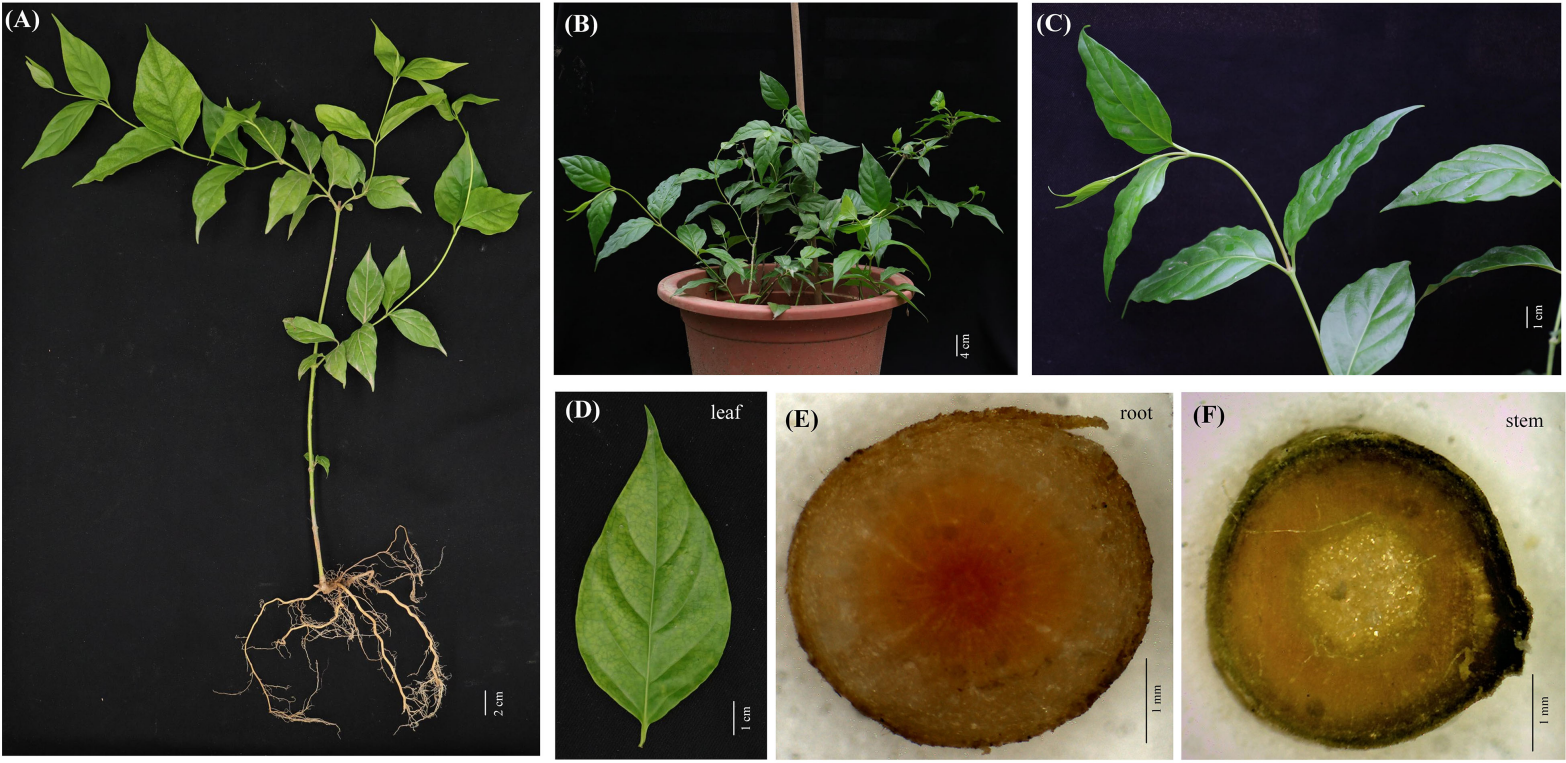
*G. elegans* plants cultured in pot. **(A–C)** The mature stage of *G. elegans*. **(D)** The leaf of *G. elegans.* **(E)** The root cross-sections. **(F)** The stem cross-section.

*G. elegans* is a woody and evergreen climber which thrives in warm and humid climates (**Figures 1A-C**). The leaves of *G. elegans* are ovate, lanceolate and verticillate (**Figure 1D**). The roots are light brown, nearly smooth and wiry. The center of the root cross section appears pink (**Figure 1E**). The stems are smooth and twining, containing a milky latex (**Figure 1F**). In this study, through LC-MS/MS we identified 26 alkaloids, including 2 gelsemine-type alkaloids, 1 koumine-type alkaloid, 12 gelsedine-type alkaloids, 2 humantenine-type alkaloids, 1 yohimbane-type alkaloid and 8 sarpagine-type alkaloids (**Figures S1**–**3** and **Table 1**). According to the structural information, most of alkaloids belonged to monoterpenoid indole alkaloids, which may derive from the precursors of tryptamine and secologanin through a series of biosynthetic processes. These alkaloids were characterized with two nitrogen atoms, and showed higher pharmacodynamics, such as analgesia, anti-tumor and anti-inflammation.

**Table 1:** Identified alkaloids in *Gelsemiume legans*.

	Alkaloid	Chemical formula	Ion pair ([M+H]^+^)	Category
1	19-(*R*)-hydroxydihydrogelsemine	C_20_H_24_N_2_O_3_	341→311	gelsemine-type
2	gelsevirine	C_21_H_24_N_2_O_3_	353→322	gelsemine-type
3	kouminol	C_20_H_24_N_2_O_2_	325→136	koumine-type
4	Nb-methylgelsedilam	C_18_H_20_N_2_O_4_	329→109	gelsedine-type
5	14-hydroxygelsenicine	C_19_H_22_N_2_O_4_	343→108	gelsedine-type
6	4,20-dehydrogelsemicine	C_20_H_24_N_2_O_4_	357→326	gelsedine-type
7	11,14-dihydroxygelsenicine	C_19_H_22_N_2_O_5_	359→108	gelsedine-type
8	gelsemicine	C_20_H_26_N_2_O_4_	359→311	gelsedine-type
9	11-hydroxygelsemicine	C_20_H_24_N_2_O_5_	373→342	gelsedine-type
10	hydroxylation of gelsemicine	C_20_H_26_N_2_O_5_	375→313	gelsedine-type
11	14-acetoxygelsenicine	C_21_H_24_N_2_O_5_	385→339	gelsedine-type
12	11-methoxydihydrogelesemine	C_21_H_28_N_2_O_5_	389→281	gelsedine-type
13	14-acetoxy-15-hydroxygelsenicine	C_21_H_24_N_2_O_6_	401→343	gelsedine-type
14	11-methoxy-19-hydroxygelselegine	C_21_H_28_N_2_O_6_	405→343	gelsedine-type
15	gelseoxazolidinine	C_23_H_28_N_2_O_6_	429→339	gelsedine-type
16	humantenine	C_21_H_26_N_2_O_3_	355→163	humantenine-type
17	11-hydroxyhumantenine	C_21_H_26_N_2_O_4_	371→325	humantenine-type
18	sempervirine	C_19_H_16_N_2_	273→245	yohimbane-type
19	dehydrokoumidine	C_19_H_20_N_20_	293→204	sarpagine-type
20	koumidine	C_19_H_22_N_2_O	295→138	sarpagine-type
21	19-(*Z*)-anhydrovobasinediol	C_20_H_24_N_2_O	309→222	sarpagine-type
22	3-hydroxykoumidine	C_19_H_22_N_2_O_2_	311→267	sarpagine-type
23	gelsemine N-oxide	C_20_H_22_N_2_O_3_	339→279	sarpagine-type
24	Na-methoxy-19-(*Z*)-anhydrovobasinediol	C_21_H_26_N_2_O_2_	339→179	sarpagine-type
25	19*E*-16-*epi*-voacarpine	C_21_H_24_N_2_O_4_	369→166	sarpagine-type
26	gelsempervine A	C_22_H_26_N_2_O_4_	383→279	sarpagine-type

### DESI based imaging pipeline for alkaloids in *G. elegans*

Among MSI-based technologies, matrix-assisted laser desorption ionization (MALDI) and DESI have been widely applied in recent years (Muller et al., 2011). MALDI-MSI requires a sample embedded in a typical matrix that can absorb laser energy and help the transfer of analytes into the gas and ionic phases. High laser precision guarantees high spatial resolution, up to 1 µm/pixel (Bjarnholt et al., 2014). However, the high matrix background noise makes MALDI-MSI unsuitable for the analysis of *m/z* less than 1000 molecules (Buscher et al., 2009). DESI-MSI has the advantages of minimal sample preparation, detection under atmospheric pressure, and small-molecule applicability (Campbell et al., 2012). Cryosectioning is a commonly used method to prepare plant tissue slices, in which freezing well quenches metabolic processes (Boughton et al., 2016). The glass slide bearing a tissue sample was placed on the mobile platform and directly analyzed by DESI-MSI. Its sample preparation is convenient, and the consumption of sample metabolites is minimal.

**Figure 2:**
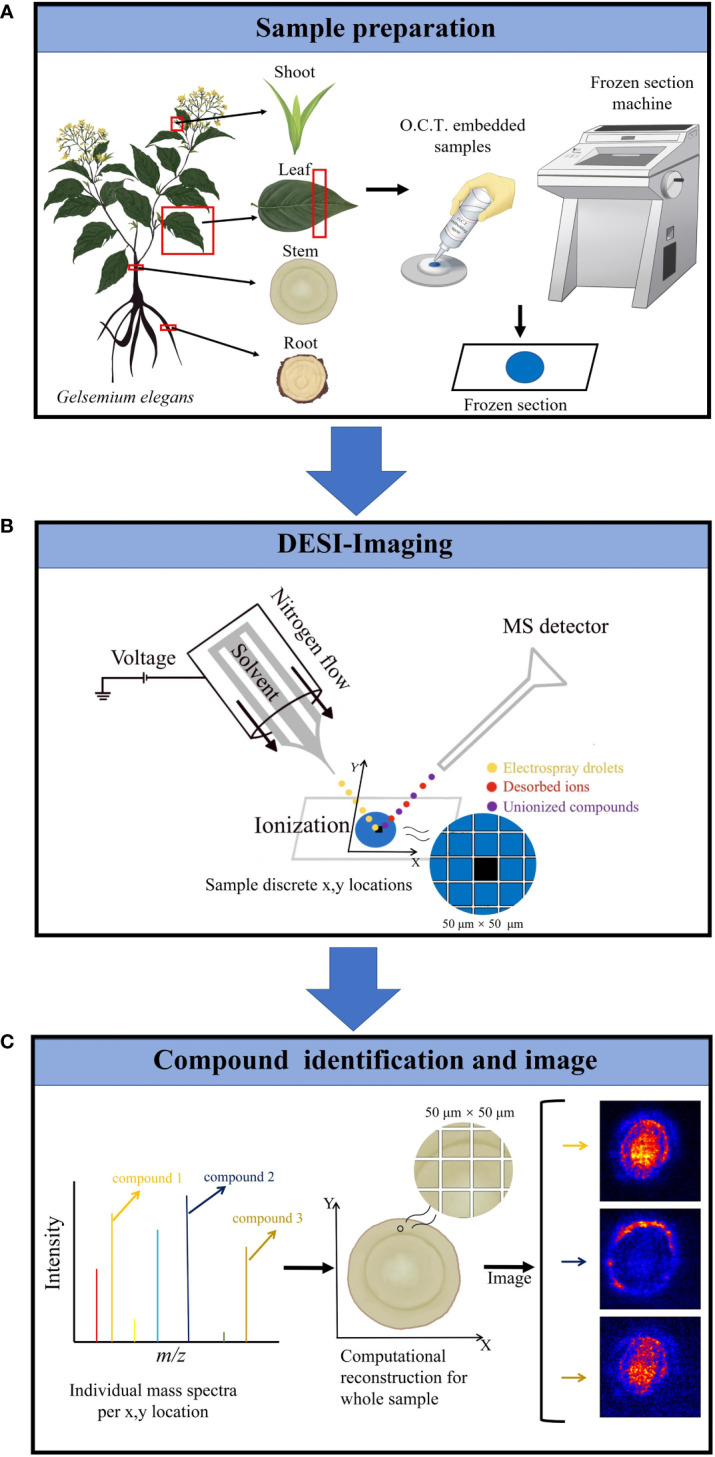
DESI-MS imaging experiment workflow. **(A)** The plant sample preparation for DESI-MSI. Frozen sections of four organ/tissue sections (roots, stems, leaves, and shoots) of *G elegans* were prepared. O.C.T. was used as the embedded agent. **(B)** The frozen sections were placed on the mobile platform of DESI-MSI. The DESI sprayer consists of a solvent emitter surrounded by a second capillary that delivers a nebulizing gas flow. Methanol in water (98:2, v/v) was used as the solvent. The DESI directs charged droplets to 50 μm × 50 μm lattice with a certain coordinate (X, Y) *via* a spray capillary, the ESI (electrospray ionization) stream impacts the extracting and ionizing analytes. Finally, the mixed droplets were ejected into the mass spectrometer. Imaging area was determined according to the lattice dimensions. **(C)** Target molecules identification and imaging. The resulting mass spectra for each coordinate (X, Y) was computationally reconstructed to form a complete dataset. The resultant reconstructed ion image represents the spatial distribution of the corresponding molecules. The acquisition setup, processing, and visualization of data were performed using HDI 1.5.

In this study, we were interested in simultaneously analyzing the spatiotemporal distributions of multiple alkaloids in organs/tissues of *G. elegans.* The molecular weight of alkaloids is generally not higher than 1000 and the structures of alkaloids are relatively stable. Due to the requirement of simultaneously analyzing multiple alkaloids in multiple organs/tissues, DESI-MSI seems to be the best choice. Therefore, DESI-MSI was employed to visualize the spatiotemporal localization of multiple alkaloids in *G. elegans* (**Figure 2**). Frozen sections of four organ/tissue sections (roots, stems, leaves, and shoots) of *G. elegans* were prepared (**Figure 2A**). The glass slide bearing a tissue sample was placed on the mobile platform. To obtain a higher image resolution, methanol in water (98:2, v/v) was used as the solvent (Manicke et al., 2008). As a key component in the DESI, the DESI sprayer consists of a solvent emitter surrounded by a second capillary that delivers a nebulizing gas flow (Wu et al., 2022a). The surface of frozen section is divided into a series of 50 μm × 50 μm lattice with a certain coordinate (X, Y). The DESI directs charged droplets to the lattice *via* a spray capillary, the ESI (electrospray ionization) stream impacts the extracting and ionizing analytes. The ions are desorbed into the gas phase and then transferred *via* an atmospheric ion transfer line into the mass spectrometer, thus enabling measurement of ions (**Figure 2B**). In the last step, the recorded mass spectra were converted into two-dimensional ion images on the certain lattice according to the coordinate (X, Y). For each spatial coordinate, the amounts of ionizable molecules present as a function of their *m/z*. The resulting mass spectra for each coordinate (X, Y) was computationally reconstructed to form a complete dataset. The resultant reconstructed ion image represented the spatial distribution of the corresponding molecules. The mass spectra were processed with Masslynx software version 4.1 and images were viewed using HDImaging version 1.4 (**Figure 2C**).

### Visualizing alkaloids locations in plant organs/tissues

To visulize the spatial distribution of alkaloids in *G. elegans*, the plant organ/tissue sections were detected by DESI-MSI in positive ionization mode. The typical DESI-MSI spectrums of alkaloids in plant organ/tissue sections were acquired (**Figure S4**). A large number of alkaloids related signals were detected in the ranges of *m/z* 100-1200. These alkaloids were confirmed by comparing the *m/z* values and MS/MS spectra with the results obtained by LC-QTOF/MS (**Table 1**).

**Figure 3:**
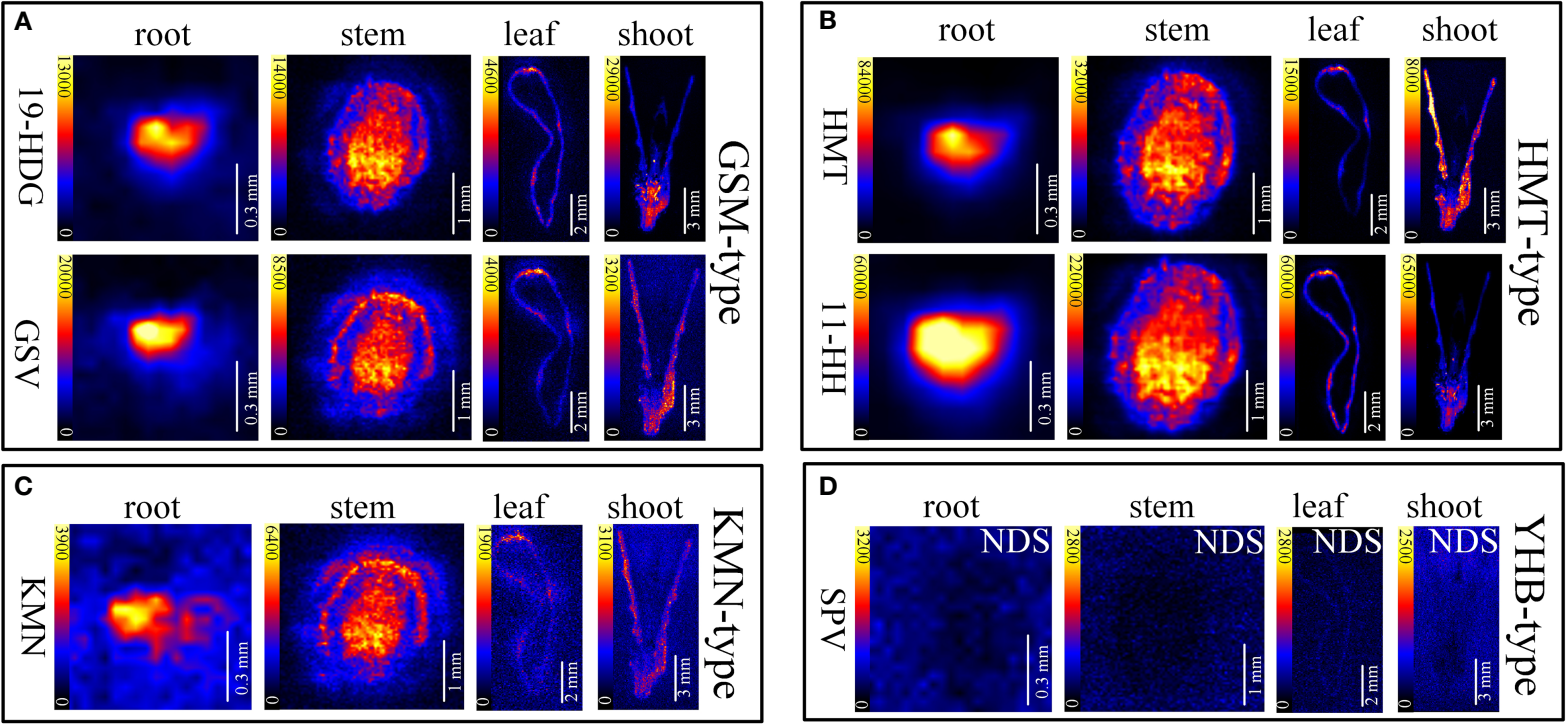
*In situ* visualization of alkaloids in sections of mature roots, stems, leaves and shoots. The color scale from dark to yellow indicates the regions of minimum (absence) and maximum relative quantification of multiple alkaloids in different organ/tissue. The relative quantifications of alkaloids were performed according to the image brightness intensities captured by the DESI-MSI. The names of the alkaloids are abbreviated. **(A)** Gelsmine-type (GSM-type) alkaloids. 19-HDG, 19-(*R*)-hydroxydihydrogelsemine (*m/z* 341.1865); GSV, gelsevirine (*m/z* 353.1865). **(B)** Humantenine-type (HMT-type) alkaloids. HMT, humantenine (*m/z* 355.2022); 11-HH, 11-hydroxyhumantenine (*m/z* 371.1971). **(C)** Koumine-type (KMN-type) alkaloids. KMN, kouminol (*m/z* 325.1916). **(D)** Yohimbane-type (YHB-type) alkaloids; SPV, sempervirine (*m/z* 273.1370). NDS means no detectable signal. Each organs/tissues had three replicates and analyzed by DESI-MSI.

**Figure 4:**
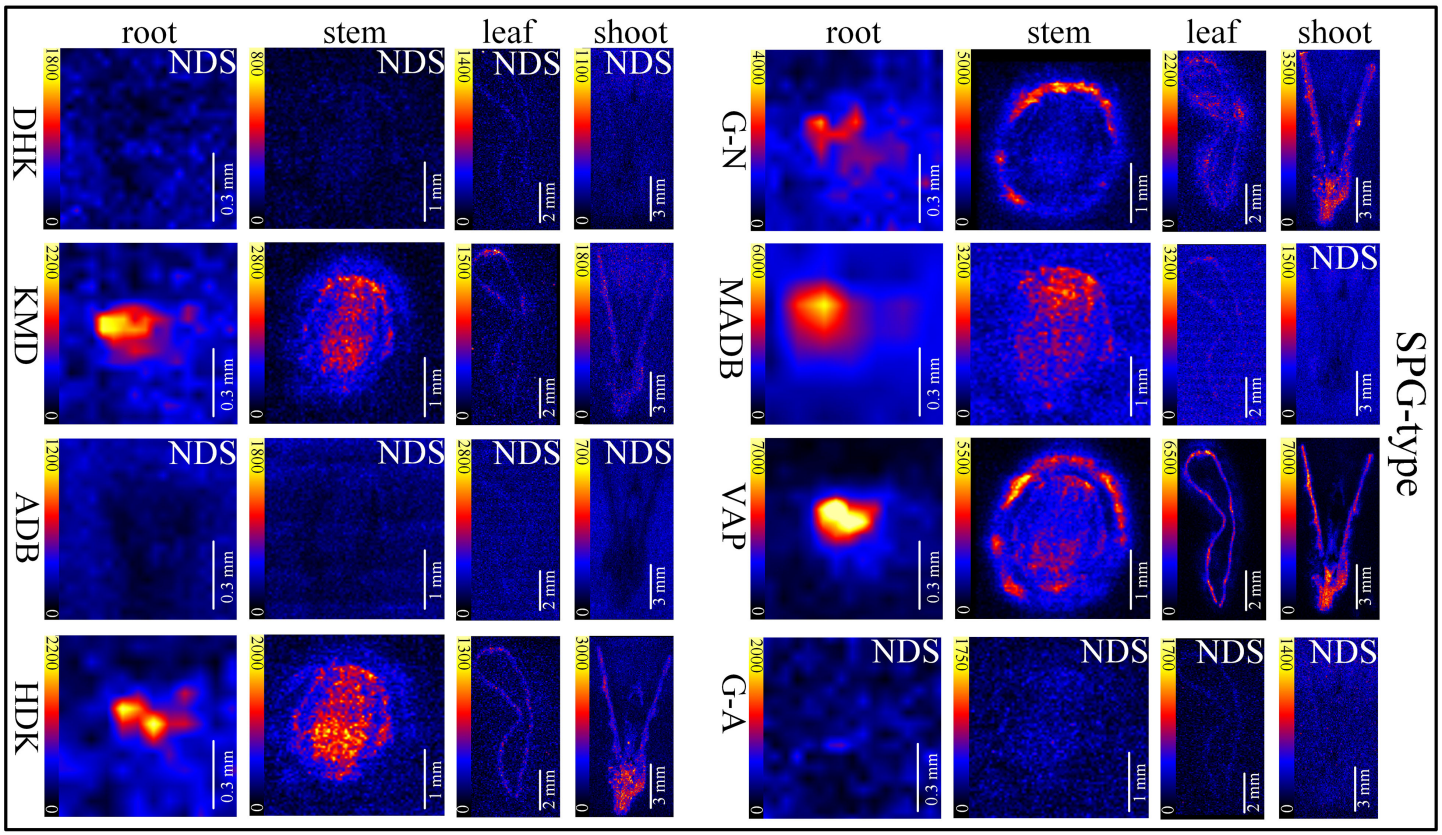
*In situ* visualization of sarpagine-type (SPG-type) alkaloids in different organ/tissue sections. NDS means no detectable signal. The names of the alkaloids are abbreviated. DHK, dehydrokoumidine (*m/z* 293.1644); KMD, koumidine (*m/z* 295.1810); ADB, 19-(*Z*)-anhydrovobasinediol (*m/z* 309.1881); HDK, 3-hydroxykoumidine (*m/z* 311.1760); G-N, gelsemine N-oxide (*m/z* 339.1709); MADB, Na-methoxy-19(*Z*)anhydrovobasinediol (*m/z* 339.2071); VAP, 19*E*-16-*epi*-voacarpine (*m/z* 369.1814); G-A, gelsempervine A (*m/z* 383.1978).

**Figure 5:**
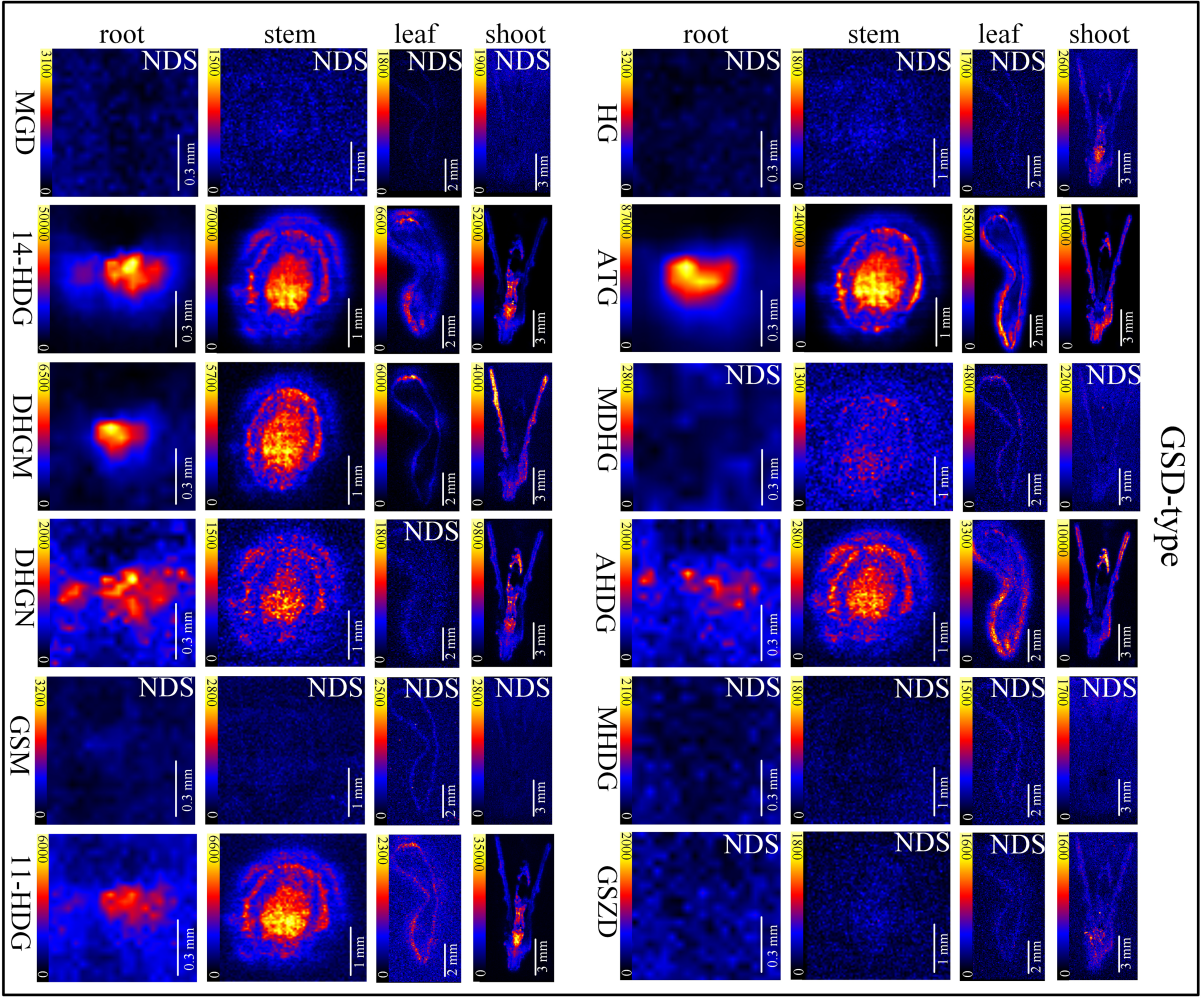
*In situ* visualization of gelsedine-type (GSD-type) alkaloids in different organ/tissue sections. NDS means no detectable signal. The names of the alkaloids are abbreviated. MGD, Nb-methylgelsedilam (*m/z* 329.1271); 14-HDG, 14-hydroxygelsenicine (*m/z* 343.1658); DHGM, 4,20-dehydrogelsemicine (*m/z* 357.1814); DHGN, 11,14-dihydroxygelsenicine (*m/z* 359.1607); GSM, gelsemicine (*m/z* 359.1951); 11-HDG:11-hydroxygelsemicine (*m/z* 373.1763); HG, hydroxylation of gelsemicine (*m/z* 375.1924); ATG, 14-acetoxygelsenicine (*m/z* 385.1763); MDHG, 11-methoxydihydrogelesemine (*m/z* 389.2085); AHDG, 14-acetoxy-15-hydroxygelsenicine (*m/z* 401.1713); MHDG, 11-methoxy-19-hydroxygelselegine (*m/z* 405.2009); GSZD, gelseoxazolidinine (*m/z* 429.2030).

*In situ* distributions of alkaloids in the sections of mature roots, stems, leaves and shoots were imaged (**Figures 3**–**5**). The microscopic pictures of frozen sections in tissues were shown in **Figure S5**. The results showed that 19 alkaloids were detected and imaged *via* DESI-MSI (**Figures 3**–**5**). 16 alkaloids were detected in the roots, all of which were located in the vascular bundle region and decreased gradually from pith to epidermis. In mature stems, 17 alkaloids were detected but their spatial distributions were not consistent. Two sarpagine-type alkaloids, gelsemine N-oxide (*m/z* 339) and 19*E*-16-*epi*-voacarpine (*m/z* 369) were mainly enriched in epidermis region, and the others displayed a similar localization pattern in roots, i.e., the 15 alkaloids were mainly located in the stem pith region and were significantly decreased in the epidermis. 16 alkaloids were detected in leaves and 17 in shoots. 16 alkaloids in leaves were mainly located in leaf veins. In shoots, most of alkaloids were distributed in young leaves and bud primordium. However, 14-hydroxygelsenicine (*m/z* 343), 11-hydroxygelsemicine (*m/z* 373) and 11,14-dihydroxygelsenicine (*m/z* 359) were distributed in bud axis which further developed into stem. Dehydrokoumidine (*m/z* 293), 19-(*Z*)-anhydrovobasinediol (*m/z* 309), gelsempervine A (*m/z* 383), sempervirine (*m/z* 273), Nb-methylgelsedilam (*m/z* 329), gelsemicine (*m/z* 359) and 11-methoxy-19-hydroxygelselegine (*m/z* 405) were not detected. In addition, some non-alkaloid constituents were also visualized by DESI-MSI, *e.g.* ferulic acid (*m/z* 195) and GEIR-1 (*m/z* 213) were detected in stems, leaves and shoots, gelsemiol (*m/z* 201) and semperoside (*m/z* 361) were detected in shoots (**Figure S6**).

### Alkaloids distributions in root at different seedling stages

The accumulation of alkaloids is both spatially and temporally. In this study, DESI-MSI was used to visualize multiple alkaloids in seedling roots of *G. elegans* respectively at 30, 60 and 90 d-age. The typical DESI-MSI spectrums of alkaloids in seeding roots were acquired (**Figure S7**). The maps captured by DESI-MSI revealed the spatial distribution of multiple alkaloids (**Figure S8**) and the image brightness intensities represented their relative contents in tissues. By using the HDI 1.5 software, the relative quantification range of alkaloids in main location was shown (**Table 2** **)**. Accordingly, 20 alkaloids were detected in *G. elegans* root at seedling stage. Along with the root development, more alkaloids were detected. For example, 14-acetoxygelsenicine (*m/z* 385) was detected in 60 d-age seedling and gelsemine N-oxide (*m/z* 339) had significant signal response in 90 d-age seedlings. In seedling roots, most of alkaloids were accumulated in the vascular bundle region. However, Nb-methylgelsedilam (*m/z* 329) were only detected in the epidermis of 60 d-age roots. Sempervirine (*m/z* 273) was enriched in the vascular bundle of 30 d-age roots, and was concentrated in the epidermis at 60 d and 90 d. Additionally, the alkaloids detected in seedling roots were not the same compared with that detected in mature roots (**Figure 3**). 14-acetoxy-15-hydroxygelsenicine (*m/z* 401) and 11,14-dihydroxygelsenicine (*m/z* 359) were detected in mature roots, but not detected in seedling roots. 19-(*Z*)-anhydrovobasinediol (*m/z* 309), gelsempervine A (*m/z* 383) and sempervirine (*m/z* 273) were only found in seedling roots.

**Table 2:** Distribution of alkaloids in seedling roots.

Category	Alkaloids	*m/z*	30 d		60 d		90 d	
			Main location	Brightness intensity	Main location	Brightness intensity	Main location	Brightness intensity
gelsemine-type alkaloids	19-(*R*)-hydroxydihydrogelsemine	341.1865	pith	7023-13812	pith	6552-9501	pith	13218-22893
	gelsevirine	353.1865	pith	1702-2922	pith	3530-5529	pith	2581-5677
koumine-type alkaloid	kouminol	325.1916	pith	1489-2219	pith	1421-3205	pith	1132-2620
gelsedine-type alkaloids	Nb-methylgelsedilam	329.1271	NDS		epidermis	439-1902	NDS	
	14-hydroxygelsenicine	343.1658	pith	601-791	pith	1480-2239	pith	4092-7726
	4,20-dehydrogelsemicine	357.1814	pith	458-859	pith	2082-2656	pith	1205-2683
	11,14-dihydroxygelsenicine	359.1607^*^	NDS		NDS		NDS	
	gelsemicine	359.1951^*^	NDS		NDS		NDS	
	11-hydroxygelsemicine	373.1763	pith	202-551	pith	1763-2678	pith	711-2982
	hydroxylation of gelsemicine	375.1924	pith	898-1341	NDS		NDS	
	14-acetoxygelsenicine	385.1763	NDS		pith	518-892	pith	2092-3890
	11-methoxydihydrogelesemine	389.2085	NDS		NDS		NDS	
	14-acetoxy-15-hydroxygelsenicine	401.1713	NDS		NDS		NDS	
	11-methoxy-19-hydroxygelselegine	405.2009	NDS		NDS		NDS	
	gelseoxazolidinine	429.2030	NDS		NDS		NDS	
humantenine-type alkaloids	humantenine	355.2022	pith	6073-12192	pith	9167-13538	pith	35577-48563
	11-hydroxyhumantenine	371.1971	pith	9569-15522	pith	13192-18574	pith	45829-81294
yohimbane-type alkaloid	sempervirine	273.1370	pith	1929-4077	epidermis	4275-21647	epidermis	1029-13829
sarpagine-type alkaloids	dehydrokoumidine	293.1644	pith	526-1131	pith	409-1083	NDS	
	koumidine	295.1810	pith	2388-4146	pith	1039-1736	pith	552-1285
	19-(*Z*)-anhydrovobasinediol	309.1881	pith	1228-2032	pith	310-1773	pith	513-2029
	3-hydroxykoumidine	311.1760	pith	2074-3817	pith	1120-1329	pith	902-1511
	gelsemine N-oxide	339.1709^*^	NDS		NDS		pith	285-1185
	Na-methoxy-19(*Z*)anhydrovobasinediol	339.2071^*^	pith	709-914	pith	519-793	pith	23-1004
	19*E*-16-*epi*-voacarpine	369.1814	pith	954-1229	pith	811-1113	pith	688-1683
	gelsempervine A	383.1978	pith	1107-1208	pith	2539-4478	epidermis	298-1438

Note: NDS means no detectable signal. _*_ means the alkaloids with very similar m/z, the ion pairs, chemical structure formulas and ion mass spectrums of these alkaloids are shown in **Table 1** and **Figures S1**, **2**. The table showed the main location of mutiple alkaloids in seedling roots. The brightness intensity range indicate relative quantification range of alkaloids in main location. The image brightness intensities were captured by DESI-MSI. performed according to the image brightness intensities captured by DESI-MSI.

### Alkaloids distributions in stem at different seedling stages

The typical DESI-MSI spectrums of alkaloids in seeding stems were acquired (**Figure S9**). 21 alkaloids were visualized in the seedling stems through DESI-MSI, and more than half of the alkaloids were detected at the later stage of seedling (**Figure S10** and **Table 3**). 10 alkaloids were detected in 30 d-age seedling, 13 alkaloids had signal response in 60 d-age seedlings, but 21 alkaloids were imaged in 90 d-age seedlings. Interestingly, the alkaloids detected in seedling stems was more than alkaloids in mature stems. A surprising discovery was that *in situ* distribution of alkaloids showed the phenomenon of transfer and diffusion along with the development and maturity of stems. In mature stems, 19*E*-16-*epi*-voacarpine (*m/z* 369) was mainly located in epidermis region (**Figure 3**), but in pith region at seedling stage. It indicated that 19*E*-16-*epi*-voacarpine gradually diffused from the inside to the outside during the growth process, and finally accumulated in epidermis region. 14-acetoxygelsenicine (*m/z* 385) and Na-methoxy-19(*Z*)anhydrovobasinediol (*m/z* 339) were enriched in pith region in 30 and 60 d-age seedlings, but diffused from pith to epidermis in 90 d-age seedlings. In addition, gelsemicine (*m/z* 359), 19-(*Z*)-anhydrovobasinediol (*m/z* 309) and gelsempervine A (*m/z* 383) were only detected in 90 d-age stems.

**Table 3:** Distribution of alkaloids in seedling stems.

Category	Alkaloids	*m/z*	30 d		60 d		90 d	
			Main location	Brightness intensity	Main location	Brightness intensity	Main location	Brightness intensity
gelsemine-type alkaloids	19-(*R*)-hydroxydihydrogelsemine	341.1865	pith	1091-2247	pith	3102-9495	pith	3016-19385
	gelsevirine	353.1865	NDS		pith	929-2394	pith	2103-15928
koumine-type alkaloid	kouminol	325.1916	NDS		pith	1211-2384	pith	1980-10920
gelsedine-type alkaloids	Nb-methylgelsedilam	329.1271	NDS		NDS		NDS	
	14-hydroxygelsenicine	343.1658	pith	311-856	pith	301-1093	pith	9988-38293
	4,20-dehydrogelsemicine	357.1814	NDS		pith	3093-4847	pith	1982-13790
	11,14-dihydroxygelsenicine	359.1607^*^	NDS		NDS		NDS	
	gelsemicine	359.1951^*^	NDS		NDS		pith	522-2847
	11-hydroxygelsemicine	373.1763	pith	233-702	pith	422-873	pith	1106-9483
	hydroxylation of gelsemicine	375.1924	pith	294-763	NDS		pith	1101-1211
	14-acetoxygelsenicine	385.1763	pith	7092-16384	pith	2122-3357	epidermis	2009-16101
	11-methoxydihydrogelesemine	389.2085	NDS		NDS		pith	272-1893
	14-acetoxy-15-hydroxygelsenicine	401.1713	NDS		NDS		epidermis	1013-38112
	11-methoxy-19-hydroxygelselegine	405.2009	NDS		NDS		NDS	
	gelseoxazolidinine	429.2030	NDS		NDS		NDS	
humantenine-type alkaloids	humantenine	355.2022	pith	1409-3745	pith	11038-17428	pith	15294-120294
	11-hydroxyhumantenine	371.1971	pith	6029-17032	pith	10294-20394	pith	8049-49338
yohimbane-type alkaloid	sempervirine	273.1370	NDS		NDS		NDS	
sarpagine-type alkaloids	dehydrokoumidine	293.1644	NDS		NDS		pith	85-1755
	koumidine	295.1810	NDS		pith	179-998	pith	982-7973
	19-(*Z*)-anhydrovobasinediol	309.1881	NDS		NDS		pith	504-4982
	3-hydroxykoumidine	311.1760	pith	2520-5921	pith	1579-3912	pith	492-5522
	gelsemine N-oxide	339.1709^*^	NDS		NDS		epidermis	97-1410
	Na-methoxy-19(*Z*)anhydrovobasinediol	339.2071^*^	pith	1262-3320	pith	412-1167	epidermis	488-4439
	19*E*-16-*epi*-voacarpine	369.1814	pith	57-782	pith	378-1259	pith	503-4928
	gelsempervine A	383.1978	NDS		NDS		pith	78-14388

Note: NDS means no detectable signal. _*_ means the alkaloids with very similar m/z, the ion pairs, chemical structure formulas and ion mass spectrums of these alkaloids are shown in **Table 1** and **Figures S1**, **2**. The table showed the main location of mutiple alkaloids in seedling stems. The brightness intensity range indicate relative quantification range of alkaloids in main location. The image brightness intensities were captured by DESI-MSI.

### Alkaloids distributions in leaf at different seedling stages

The typical DESI-MSI spectrums of alkaloids in seeding leaves were acquired (**Figure S11**). In seedling leaves, many alkaloids showed different distribution patterns (**Figure S12** and **Table 4**). 16 alkaloids were detected in seedling leaves (**Figure 3**). 14-acetoxy-15-hydroxygelsenicine (*m/z* 401) and Na-methoxy-19(*Z*) anhydrovobasinediol (*m/z* 339) were respectively detected in leaf of 60 and 90 d-age seedling. As results, alkaloids were mainly concentrated in mesophyll at the early stage of seedling leaves, but they gradually enriched in leaf veins along with the development and eventually accumulates in large quantities at the later stage of seedling leaves. However, 14-acetoxy-15-hydroxygelsenicine (*m/z* 401) distribution was scattered in the leaves of 90 d-age seedling. 4 alkaloids were imaged in 30 d-age seedling, and no signal response in 60 d-age seedling, but they were surprisingly detected again in the leaves of 90 d-age seedling.

**Table 4:** Distribution of alkaloids in seedling leaves.

Category	Alkaloids	*m/z*	30 d		60 d		90 d	
			Main location	Brightness intensity	Main location	Brightness intensity	Main location	Brightness intensity
gelsemine-type alkaloids	19-(*R*)-hydroxydihydrogelsemine	341.1865	mesophyll	767-4648	mesophyll	438-1711	vein	1039-8924
	gelsevirine	353.1865	mesophyll	211-1455	mesophyll	393-1503	vein	514-2948
koumine-type alkaloid	kouminol	325.1916	mesophyll	83-1575	NDS		vein	286-2855
gelsedine-type alkaloids	Nb-methylgelsedilam	329.1271	NDS		NDS		NDS	
	14-hydroxygelsenicine	343.1658	mesophyll	29-692	mesophyll	93-877	vein	449-2983
	4,20-dehydrogelsemicine	357.1814	mesophyll	332-1439	mesophyll	474-9469	vein	2103-13957
	11,14-dihydroxygelsenicine	359.1607^*^	NDS		NDS		NDS	
	gelsemicine	359.1951^*^	NDS		NDS		NDS	
	11-hydroxygelsemicine	373.1763	mesophyll	83-1019	mesophyll	309-893	vein	77-1059
	hydroxylation of gelsemicine	375.1924	NDS		NDS		NDS	
	14-acetoxygelsenicine	385.1763	mesophyll	1032-7093	mesophyll	694-2355	vein	1112-11948
	11-methoxydihydrogelesemine	389.2085	NDS		NDS		NDS	
	14-acetoxy-15-hyd roxygelsenicine	401.1713	NDS		mesophyll	94-683	mesophyll	182-1055
	11-methoxy-19-hydroxygelselegine	405.2009	NDS		NDS		NDS	
	gelseoxazolidinine	429.2030	NDS		NDS		NDS	
humantenine-type alkaloids	humantenine	355.2022	mesophyll	1232-14429	mesophyll	3193-9523	vein	5016-69301
	11-hydroxyhumantenine	371.1971	mesophyll	4034-11074	mesophyll	3815-11093	vein	4022-18192
yohimbane-type alkaloid	sempervirine	273.1370	NDS		NDS		NDS	
sarpagine-type alkaloids	dehydrokoumidine	293.1644	NDS		NDS		NDS	
	koumidine	295.1810	NDS		NDS		vein	419-1672
	19-(*Z*)-anhydrovobasinediol	309.1881	mesophyll	84-1410	NDS		vein	526-1611
	3-hydroxykoumidine	311.1760	mesophyll	1432-4783	NDS		vein	392-1492
	gelsemine N-oxide	339.1709^*^	mesophyll	133-691	NDS		vein	189-1782
	Na-methoxy-19(*Z*)anhydrovobasinediol	339.2071^*^	NDS		NDS		vein	312-2188
	19*E*-16-*epi*-voacarpine	369.1814	mesophyll	392-1321	mesophyll	343-1511	vein	179-2801
	gelsempervine A	383.1978	NDS		NDS		NDS	

Note: NDS means no detectable signal. _*_ means the alkaloids with very similar m/z, the ion pairs, chemical structure formulas and ion mass spectrums of these alkaloids are shown in **Table 1** and **Figures S1**, **2**. The table showed the main location of mutiple alkaloids in seedling leaves. The brightness intensity range indicate relative quantification range of alkaloids in main location. The image brightness intensities were captured by DESI-MSI.

## Discussion

### Capabilities and applications of MSI in metabolomics

MSI has been developed to understand the spatial distribution of small organic molecules in organisms, and has been successfully applied to a variety of plants, including *Hypericum perfortum* roots (Tocci et al., 2018), *Ginkgo biloba* leaves (Li et al., 2018), *Vitex agnus-castus* fruits and leaves (Heskes et al., 2018) and strawberry fruits (Enomoto et al., 2018). MSI analysis enables spatial acquisition of targeted or untargeted metabolism data. MSI permits the determination of exactly where the target metabolites accumulate because of its higher resolution and accuracy. Recently, spatial metabolomics was developed based on MSI, which has greatly accelerated the development of biomedicine (Yen et al., 2014). This method was performed to study the distribution of key flavonoids involved in various synthesis pathways in mint leaves (Freitas et al., 2019). Through spatial metabolomics based on MSI, the localization of asparaptine A was identified in *Asparagus officinalis* (Nakabayashi et al., 2021). Spatial metabolomics was employed to simultaneously determine the spatial localization and distribution patterns of endogenous molecules in plant tissues and provide a theoretical basis for understanding the synthesis and interactions of multiple components (Palla et al., 2022).

### DESI-MSI for visualizing spatiotemporal distribution of multiple alkaloids in *Gelsemium elegans*

Since the relationship between metabolites and their spatial distribution in plants attracts research interests (Moreno-Pedraza et al., 2019), DESI was successfully applied to the detection of alkaloids. In recent years, HPTLC-DESI-MS^n^ was used to identify 13 aporphine and 4 benzylisoquinoline-type alkaloids in *Ocotea spixiana* (Conceicao et al., 2020). The quantitative imaging of 7 *Uncaria* alkaloids in rat brains using DESI-MSI was also performed (Gao et al., 2022). DESI-MSI was also used to visualize the spatial distribution of 63 metabolites in *Salvia miltiorrhiza*, and the complementary data obtained from the metabolomics coupled with mass spectrometry imaging enabled the identification of key reactions involved in flavonoid biosynthesis (Tong et al., 2022). Therefore, DESI-MSI is an useful tool to analyze the relationship between the spatial distribution and relative content of a specific compound. The authors’ group has previously applied DESI-MSI and visualized the spatial distribution of three alkaloids (gelsemine, koumine, and gelsenicine) in *G. elegans* (Wu et al., 2022b). In this study, *in situ* distribution of multiple alkaloids in organ/tissue sections of *G. elegans* at different growth stages were simultaneously visualized by DESI-MSI. 26 alkaloids in *G. elegans* were analyzed by LC-MS/MS, and the precursor ions, product ions and chemical formulas of these alkaloids were presented (**Table 1**). Among a total of 23 alkaloids detected in roots, stems, leaves and shoots of *G. elegans* by DESI-MSI, 19 alkaloids were found to exhibit specific spatiotemporal distribution *in planta* (**Figure 3**).

In mature roots, 16 alkaloids were located in vascular bundle region, and were decreased gradually from pith to epidermis. However, research on *Rauvolfia tetraphylla* showed that the spatial distribution of three alkaloids in roots mainly accumulated in epidermis (Kumara et al., 2019), indicating the *in-situ* distribution of alkaloids in roots of different medical plants is tissue specific. In mature stems, 17 alkaloids were detected and most of them were distributed in the pith region. However, sarpagine-type alkaloids such as gelsemine N-oxide and 19*E*-16-*epi*-voacarpine were mainly enriched in epidermis region. Sarpagine-type alkaloids serve as the precursors for the more complex ajmaline- and koumine- type indole alkaloids, and exhibit antimalarial activity (Chen et al., 2022). The accumulation of alkaloids is both spatially and temporally. Multiple alkaloids in roots, stems and leaves at different seedling stages of *G. elegans* were visualized by DESI-MSI (**Figures S7**–**12**; **Tables 2** **–** **4**). Moreover, the relative quantification (RQ) of alkaloids were performed according to the image brightness intensities captured by the DESI-MSI. The relative content of most alkaloids increased along with the growth of *G. elegans.* As many as 20, 21 and 16 alkaloids were respectively detected in seedling roots, stems and leaves. In seedling roots, 14-acetoxygelsenicine and gelsemine N-oxide showed spatial distribution from 60 d and 90 d, respectively. In seedling leaves, 14-acetoxy-15-hydroxygelsenicine and Na-methoxy-19-(*Z*)-anhydrovobasinediol were detected from 60 d and 90 d. In seedling stems, the accumulation time points of alkaloids were found to be inconsistent, 10 and 13 alkaloids had signal response respectively at 30 d, 60 d, and the later stage of seedling. Several secondary metabolites have been reported to accumulate in developing seeds, presumably confer resistance against abiotic and biotic stress during seed dispersal and germination (Patrick and Offler, 2001; Dyer et al., 2001; Alves et al., 2007). Secondary metabolites are either synthesized *de novo* in the seed from available precursors or are transported from elsewhere in the plant. In the present study, although it is hard to tell whether the alkaloids detected in seedlings at 30 d are synthesized locally or not, it is sure that the alkaloids detected at the later stage was produced by a series of enzymatic reactions during plant development. In either case, it will be interesting to examine their spatial and temporal pattern of accumulation. Another surprising finding was the spatial distribution of multiple alkaloids changed significantly along with the stem development, 14-acetoxygelsenicine and Na-methoxy-19-(*Z*)-anhydrovobasinediol were enriched in vascular bundle region at the early stage of seedling and then diffused to epidermis at 90 d. 19*E*-16-*epi*-voacarpine was mainly located in vascular bundle region of seedling stems, and then diffused to epidermis region of mature stems. Since the biosynthesis and storage of plant metabolites are highly regulated, it is possible that these alkaloids localized in stems at the early stage will play roles in stress resistance at different growth stages or the alkaloids transportation to the epidermis from other parts are involved. Thus the spatial-temporal distribution of these alkaloids would be precisely regulated in stress responses.

The spatial metabolomics is a developing sub-branch of metabolomics rarely employed in medical plant studies, especially the plant alkaloids research. Only a few studies in plants have been previously reported including the spatial metabolome (monoterpene and paeonol glycosides, tannins, flavonoids, saccharides and lipids) of *Paeonia suffruticosa* and *Paeonia lactiflora* roots by MALDI-MSI (Li et al., 2021), the spatial metabolome (flavonoids, ginkgolic acids, cardanols, saccharides, phospholipids and chlorophylls) of *Ginkgo biloba* by MALDI and LDI-MSI (Li et al., 2018). Using DESI-MSI method, we successfully visualized the spatial distribution for as more as 26 alkaloids in different *G. elegans* tissues at different growth stages, and analyzed the spatial transport of specific alkaloid. In addition to the visualization of spatial distribution of alkaloids in plant tissues, this study also provides robust data for further constructing spatial omics frameworks for multiple alkaloids in *G. elegans*, which will greatly promote DESI-MSI application in plants.

## Conclusion

The DESI-MSI technique provides an efficient approach to directly visualize the target molecules *in situ* in plants without the extra extraction and processing. In this study, 23 alkaloids were visualized in roots, stems and leaves at seedling stage and 19 alkaloids were observed at mature stage by using DESI-MSI. Among them, 16 alkaloids were distributed in vascular bundle region of mature roots, 15 alkaloids were mainly located in the pith region of mature stems and 2 alkaloids were enriched in epidermis region of mature stems. In addition, 17 alkaloids were detected in shoots and 16 alkaloids were detected in mature leaf veins. Interestingly, along with the development process of *G. elegans*, *in situ* distribution showed that multiple alkaloids in tissues may undergo diffusion and transfer. Overall, our work indicated DESI-MSI is a promising spatial omics technique for visualizing the spatiotemporal distribution of alkaloids in plants.

## Data availability statement

The original contributions presented in the study are included in the article/**Supplementary Material**. Further inquiries can be directed to the corresponding authors.

## Author contributions

Z-HW and R-ZW developed the sample preparation and MSI method, and compiled the manuscript. YS interpreted the results and drafted the manuscript. Z-LS supported the method development and the data analysis. L-TX acquired funding, supervised the project and revised the manuscript. All authors contributed to the article and approved the submitted version.

## Funding

This study was supported by the financial support from the National Natural Science Foundation of China (grant number: 91317312 and 31871714), and Provincial Natural Science Foundation of Hunan (grant number: 2022JJ30294).

## Acknowledgments

We are very grateful to Zhihong Gong (Waters Corporation, Shanghai) for his technical support during DESI experiments, Zhaoying Liu (Hunan Agricultural University, Changsha) for providing seeds of *Gelsemium elegans*, and Yaqi Liu (University of the Arts London, London) for image processing assistance.

## Conflict of interest

The authors declare that the research was conducted in the absence of any commercial or financial relationships that could be construed as a potential conflict of interest.

## Publisher’s note

All claims expressed in this article are solely those of the authors and do not necessarily represent those of their affiliated organizations, or those of the publisher, the editors and the reviewers. Any product that may be evaluated in this article, or claim that may be made by its manufacturer, is not guaranteed or endorsed by the publisher.
