# Supplementary material for: A mass spectrometry imaging approach on spatiotemporal distribution of multiple alkaloids in Gelsemium elegans
Source: Front Plant Sci. 2022 Nov 18;13:1051756. doi: 10.3389/fpls.2022.1051756 (PMC9718364; doi:10.3389/fpls.2022.1051756)
Supplement: Supplementary file 1 [file DataSheet_1.pdf]

Supplementary Materials

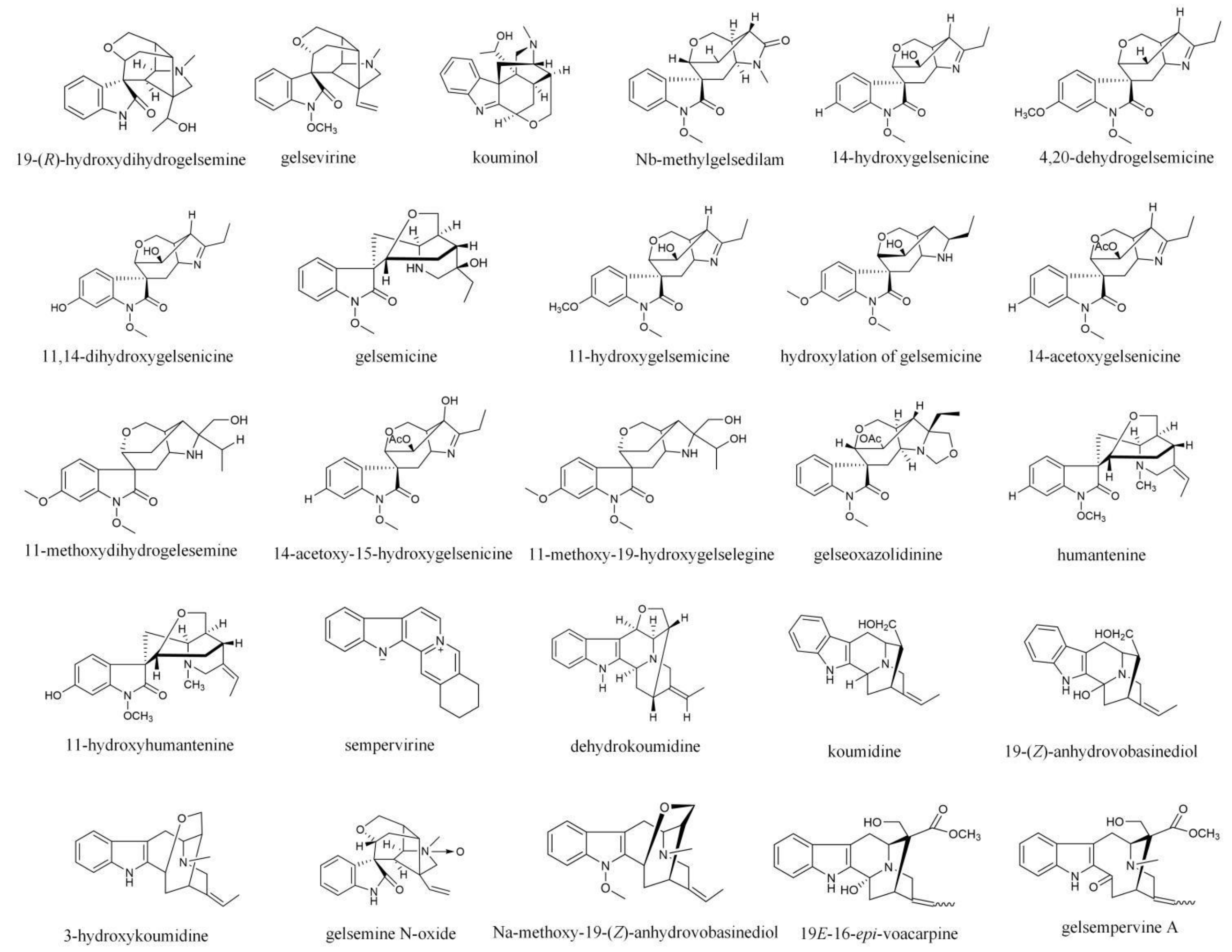

Figure S1. The chemical structure of 26 alkaloids in *G. elegans*.

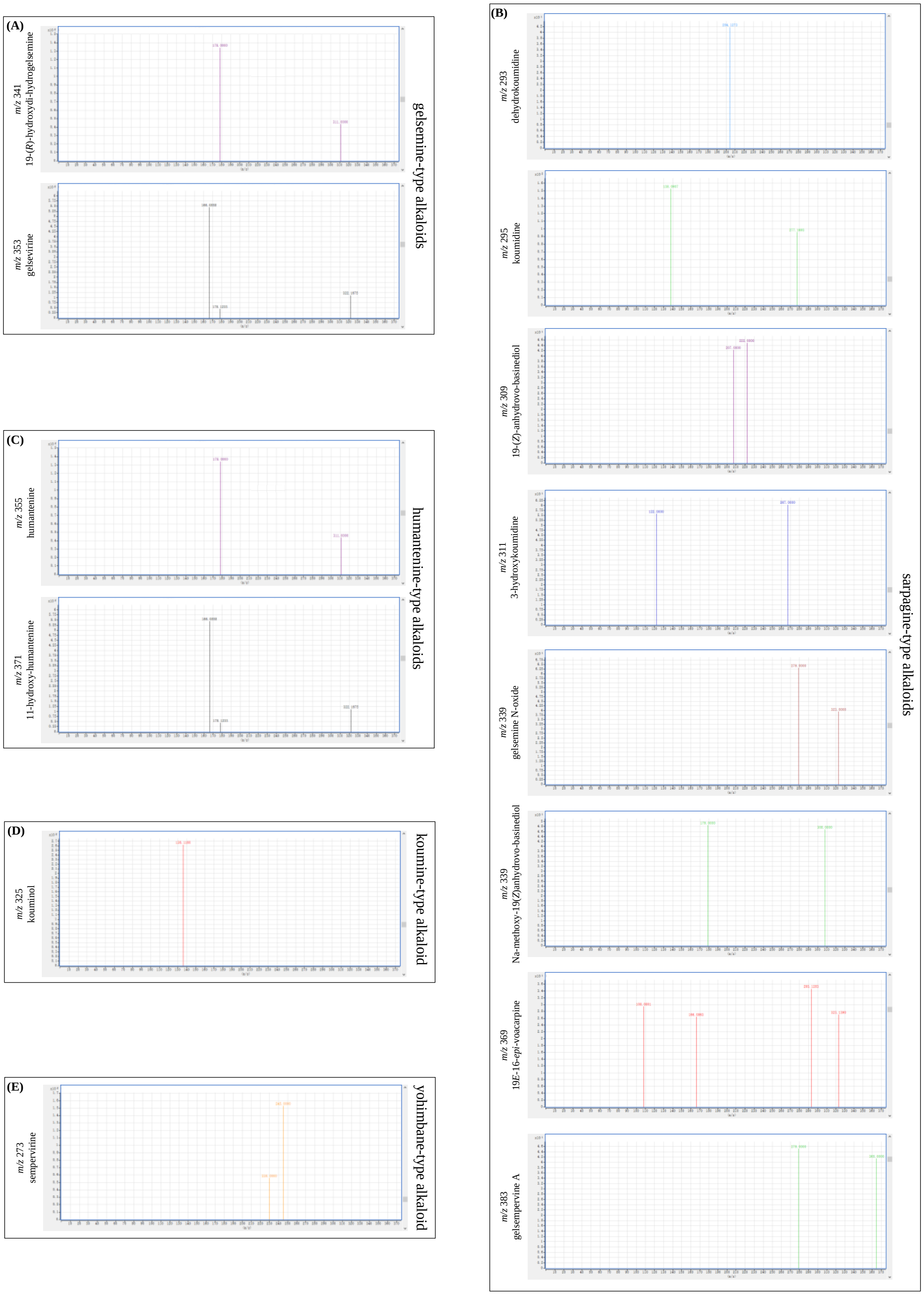

Figure S2. Ion mass spectrums of alkaloids by LC-MS/MS. **(A)** Ion mass spectrums of gelsmine-type alkaloids by LC-MS/MS. **(B)** Ion mass spectrums of sarpagine-type alkaloids by LC-MS/MS. **(C)** Ion mass spectrums of humantenine-type alkaloids by LC-MS/MS. **(D)** Ion mass spectrums of koumine-type alkaloids by LC-MS/MS. **(E)** Ion mass spectrums of yohimbane-type alkaloids by LC-MS/MS.

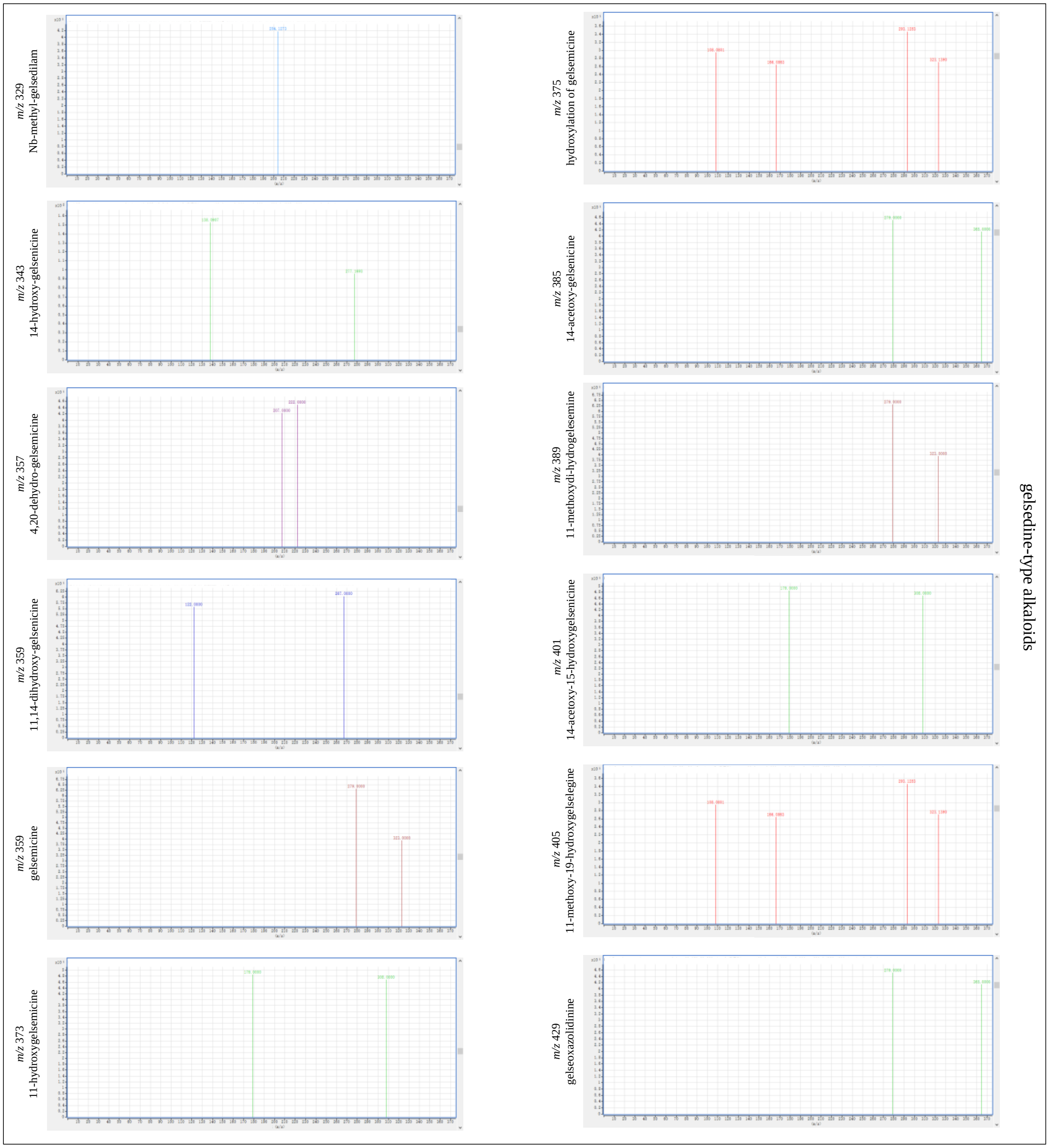

Figure S3. Ion mass spectrums of gelsedine-type alkaloids by LC-MS/MS.

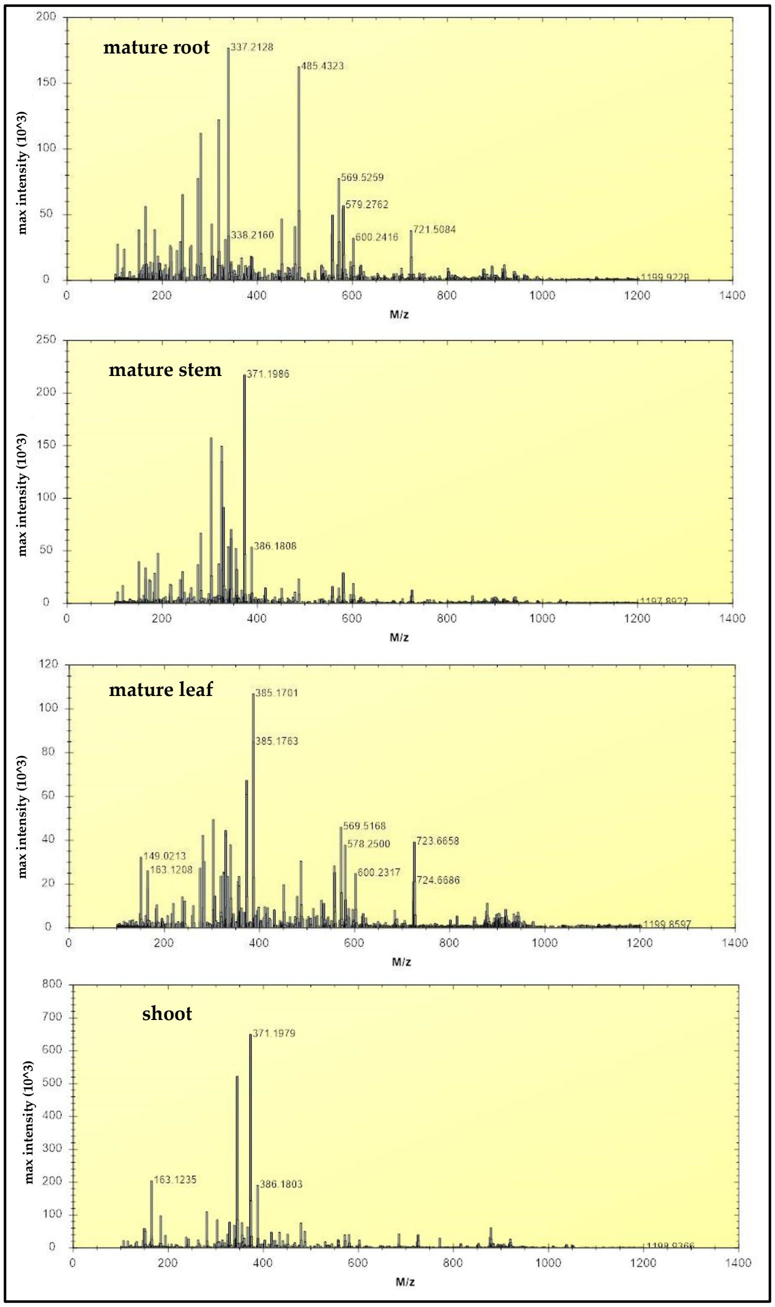

Figure S4. The typical DESI-MSI spectrums acquired from plant organ/tissue sections in positive ionization mode.

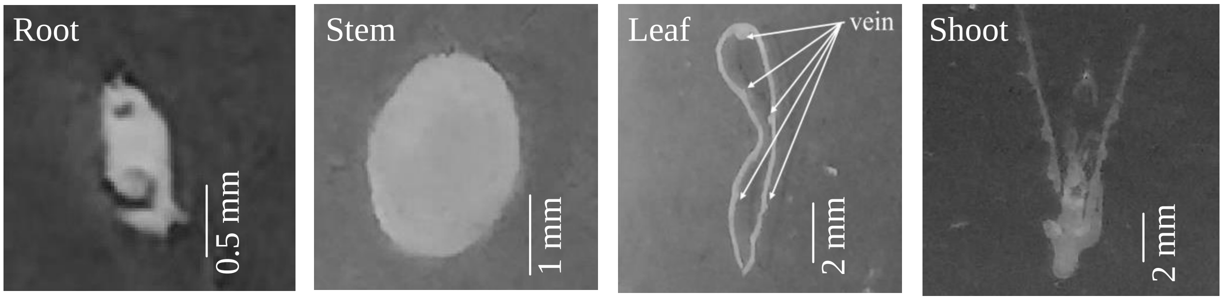

Figure S5. The microscopic pictures of frozen sections of mature organs/tissues.

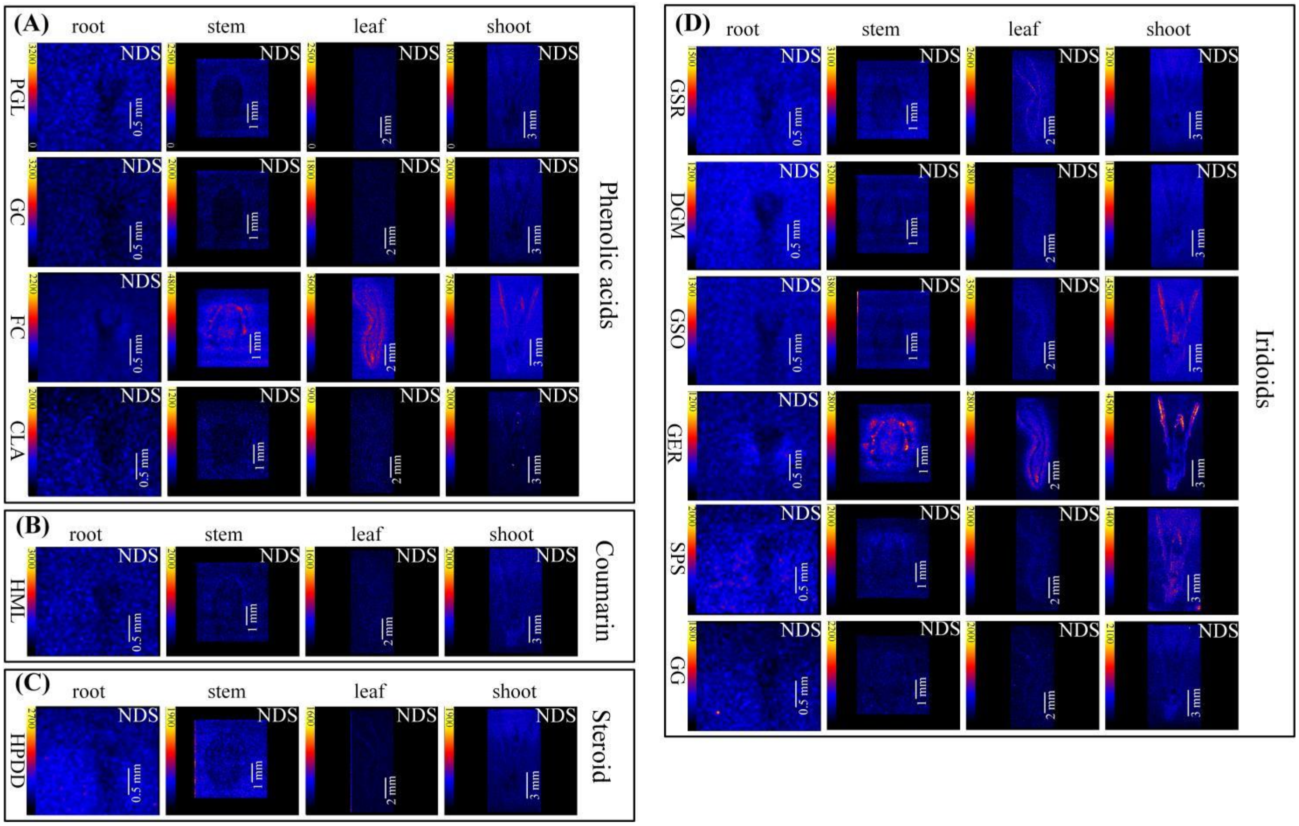

Figure S6. Spatial distribution of non-alkaloids in mature organs/tissues. The names of the non-alkaloids are abbreviated. **(A)** Phenolic acids. PGL: pyrogallol (*m/z* 127); GC: gallic acid (*m/z* 171); FC: ferulic acid (*m/z* 195); CLA: 1-O-cffeoy lquinic acid (*m/z* 355). **(B)** Coumarin. HML: 6-hydroxy-7-methoxy-lcoumarin (*m/z* 193). **(C)** Steroid. HPDD: 12-hydroxy-pregn-4,16-diene-3,20-dione (*m/z* 329). **(D)** Iridoids. GSR: GSIR-1 (*m/z* 183); DGM: 7-deoxygelsemid (*m/z* 197); GSO: gelsemiol (*m/z* 201); GER: GEIR-1 (*m/z* 201); SPS: semperoside (*m/z* 361); GG: gelsemiol-3-glucoside (*m/z* 363). NDS means no detectable signal.

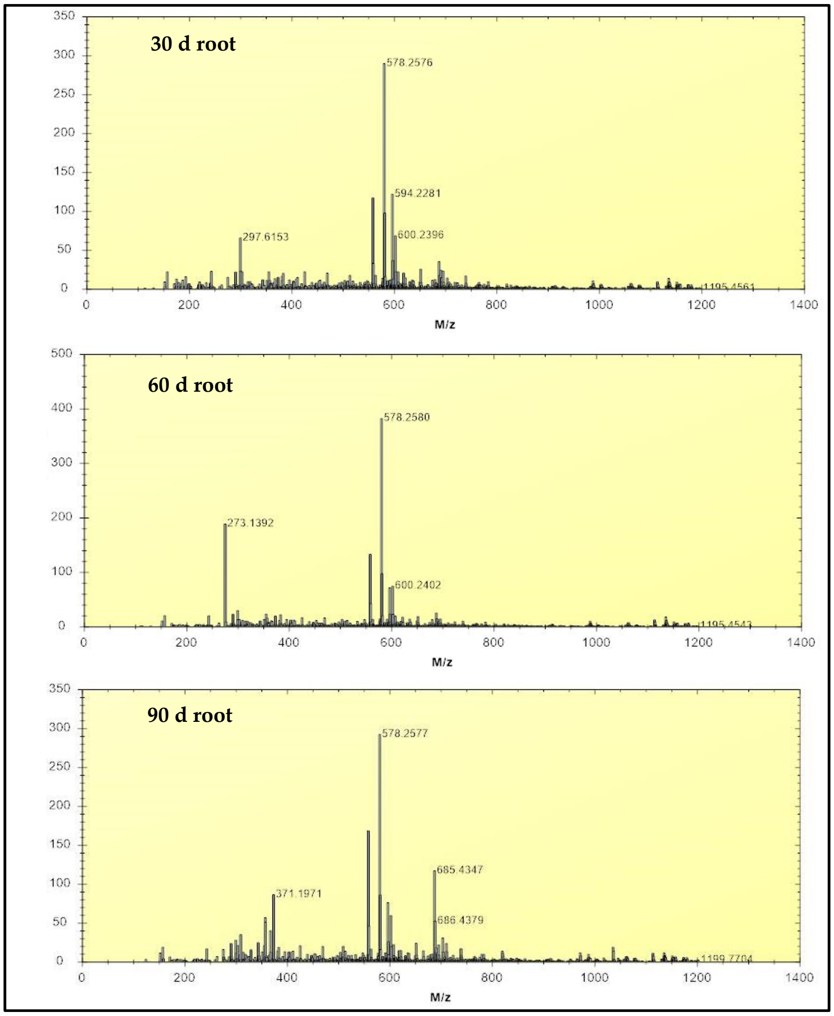

Figure S7. The DESI-MSI spectrums of seedling roots.

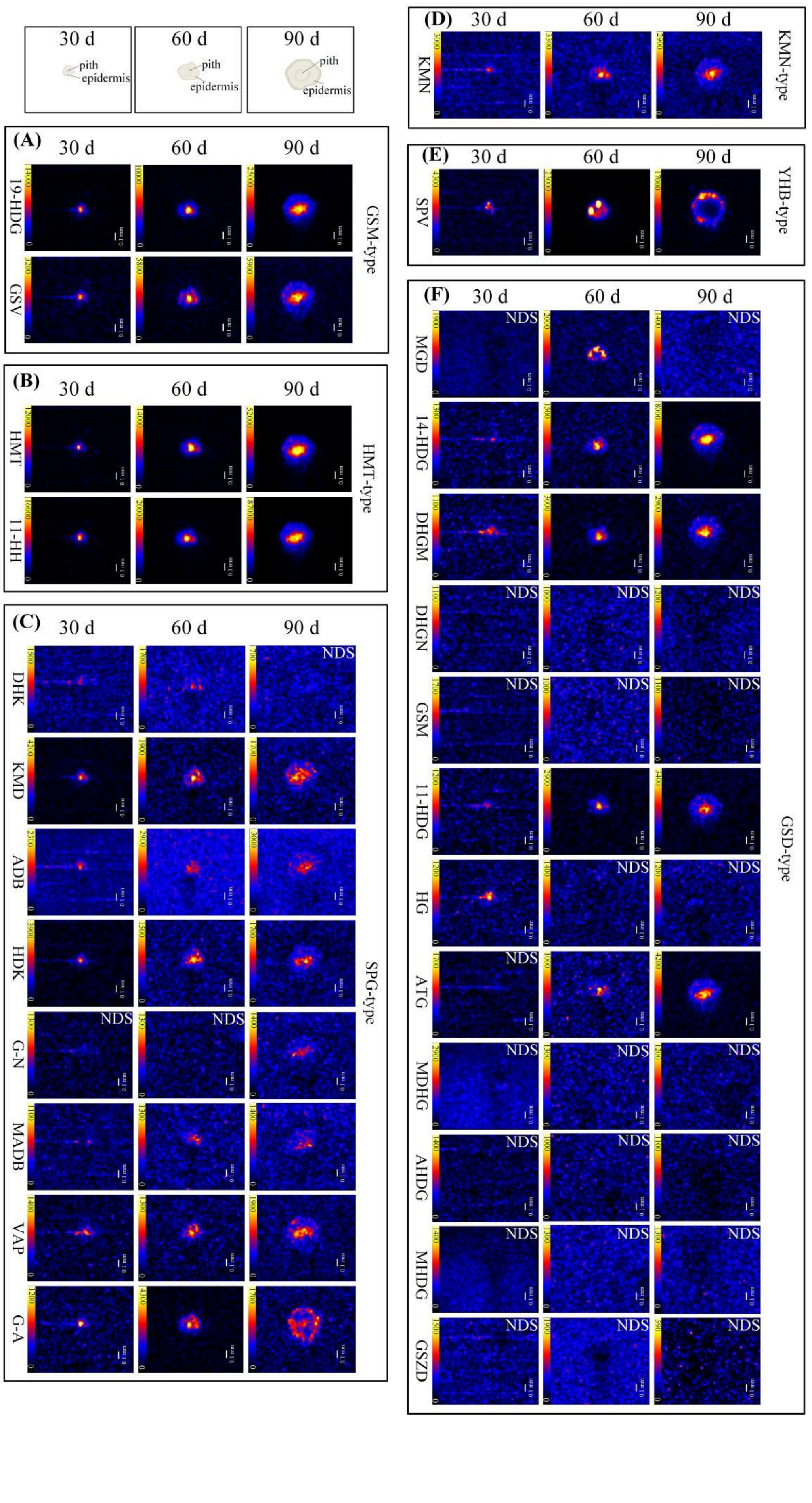
30 d
60 d
90 d
pith
epidermis
(A)
19-HDG
GSV
GSM-type
(B)
HMT
11-HH
HMT-type
(C)
DHK
KMD
ADB
HDK
G-N
MADB
VAP
G-A
SPG-type
NDS
(D)
KMN
KMN-type
(E)
SPV
YHB-type
(F)
MGD
14-HDG
DHGM
DHGN
GSM
11-HDG
HG
ATG
MDHG
AHDG
MHDG
GSZD
GSD-type
0.1 mm

Figure S8. *In situ* visualization of alkaloids in seedling roots. The sizes of the pattern photos of seedling root cross sections correspond to the sizes of the DESI-images. NDS means no detectable signal. Each organs/tissues had three replicates and analyzed by DESI-MSI. The names of the alkaloids are abbreviated. **(A)** Gelsmine-type (GSM-type) alkaloids. 19-HDG: 19-(*R*)-hydroxydihydrogelsemine (*m/z* 341.1865); GSV: gelsevirine (*m/z* 353.1865). **(B)** Humantenine-type (HMT-type) alkaloids. HMT: humantenine (*m/z* 355.2022); 11-HH: 11-hydroxyhumantenine (*m/z* 371.1971). **(C)** Sarpagine-type (SPG-type) alkaloids. DHK: dehydrokoumidine (*m/z* 293.1644); KMD: koumidine (*m/z* 295.1810); ADB: 19-(*Z*)-anhydrovobasinediol (*m/z* 309.1881); HDK: 3-hydroxykoumidine (*m/z* 311.1760); G-N: gelsemine N-oxide (*m/z* 339.1709); MADB: Na-methoxy-19(*Z*)anhydrovobasinediol (*m/z* 339.2071); VAP: 19*E*-16-*epi*-voacarpine (*m/z* 369.1814); G-A: gelsempervine A (*m/z* 383.1978). **(D)** Koumine-type (KMN-type) alkaloids. KMN: kouminol (*m/z* 325.1916). **(E)** Yohimbane-type (YHB-type) alkaloids. SPV: sempervirine (*m/z* 273.1370). **(F)** Gelsedine-type (GSD-type) alkaloids. MGD: Nb-methylgelsedilam (*m/z* 329.1271); 14-HDG: 14-hydroxygelsenicine (*m/z* 343.1658); DHGM: 4,20-dehydrogelsemicine (*m/z* 357.1814); DHGN: 11,14-dihydroxygelsenicine (*m/z* 359.1607); GSM: gelsemicine (*m/z* 359.1951); 11-HDG:11-hydroxygelsemicine (*m/z* 373.1763); HG: hydroxylation of gelsemicine (*m/z* 375.1924); ATG: 14-acetoxygelsenicine (*m/z* 385.1763); MDHG: 11-methoxydihydrogelesemine (*m/z* 389.2085); AHDG: 14-acetoxy-15-hydroxygelsenicine (*m/z* 401.1713); MHDG: 11-methoxy-19-hydroxygelselegine (*m/z* 405.2009); GSZD: gelseoxazolidinine (*m/z* 429.2030).

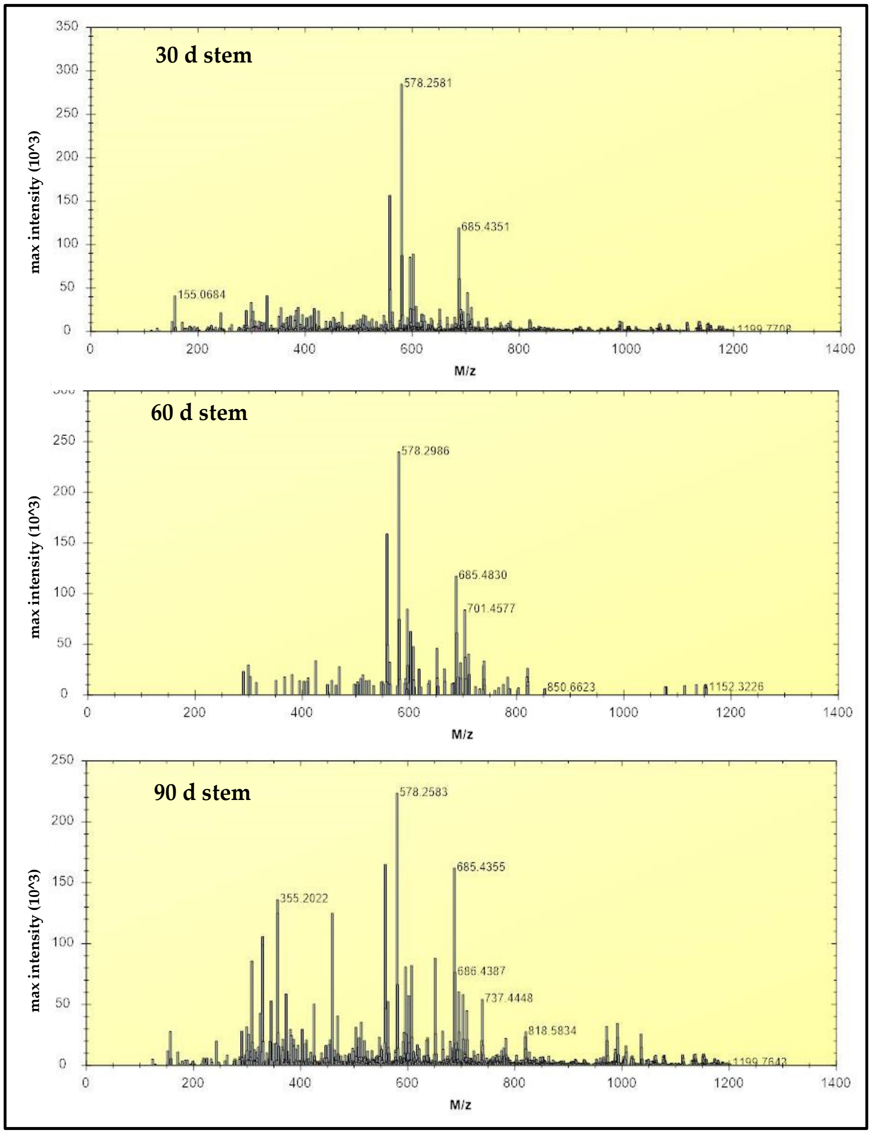

Figure S9. The DESI-MSI spectrums of seedling stems.

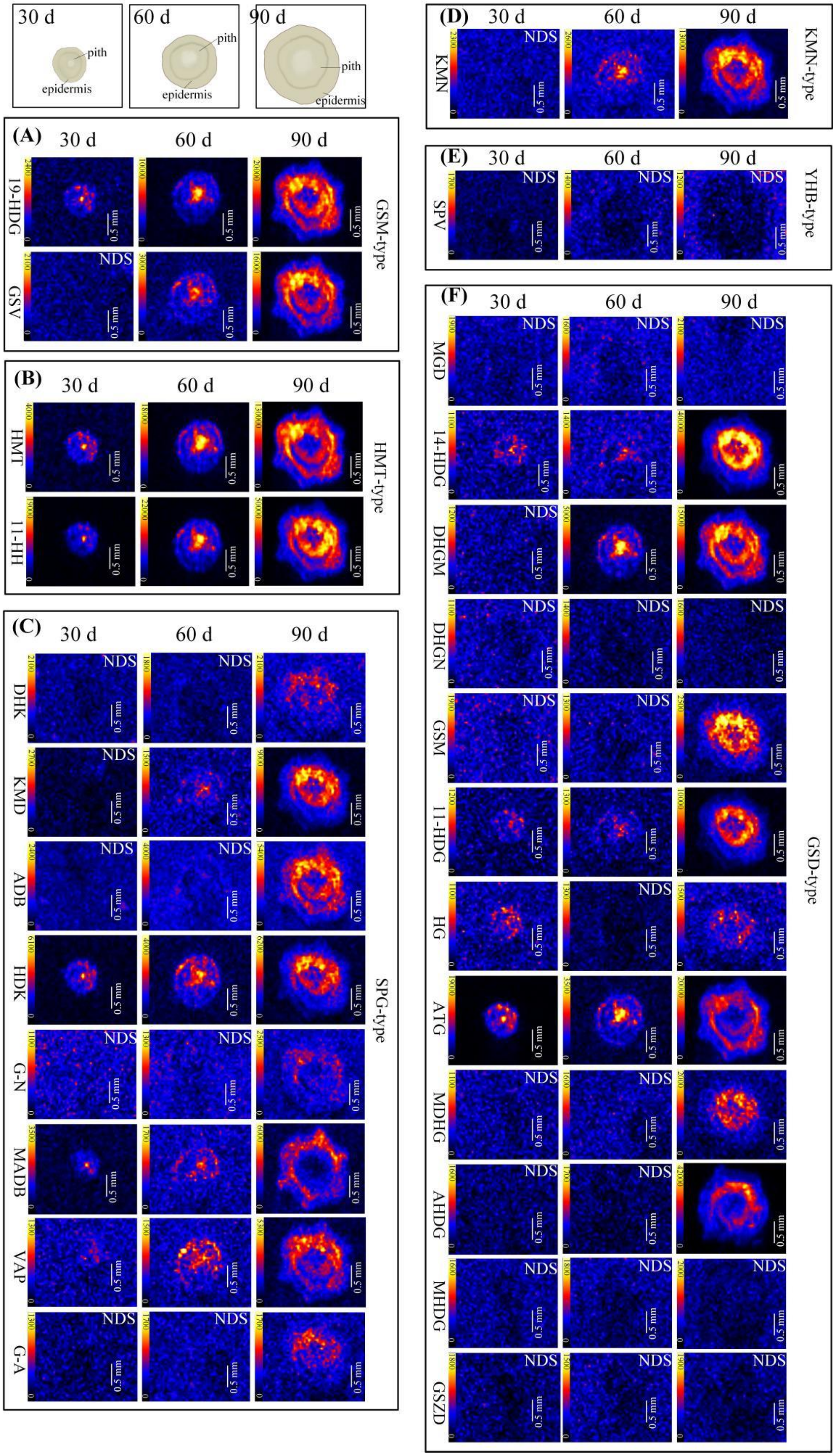
30 d
60 d
90 d
pith
epidermis
(A)
19-HDG
GSV
GSM-type
(B)
HMT
11-HH
HMT-type
(C)
DHK
KMD
ADB
HDK
G-N
MADB
VAP
G-A
SPG-type
(D)
KMN
KMN-type
(E)
SPV
YHB-type
(F)
MGD
14-HDG
DHGM
DHGN
GSM
11-HDG
HG
ATG
MDHG
AHDG
MHDG
GSZD
GSD-type
NDS
0.5 mm

Figure S10. *In situ* visualization of alkaloids in seedling stems. The sizes of the pattern photos of seedling stem cross scetions correspond to the sizes of the DESI-images. NDS means no detectable signal. Each organs/tissues had three replicates and analyzed by DESI-MSI. The names of the alkaloids are abbreviated. **(A)** Gelsmine-type (GSM-type) alkaloids. 19-HDG: 19-(*R*)-hydroxydihydrogelsemine (*m/z* 341.1865); GSV: gelsevirine (*m/z* 353.1865). **(B)** Humantenine-type (HMT-type) alkaloids. HMT: humantenine (*m/z* 355.2022); 11-HH: 11-hydroxyhumantenine (*m/z* 371.1971). **(C)** Sarpagine-type (SPG-type) alkaloids. DHK: dehydrokoumidine (*m/z* 293.1644); KMD: koumidine (*m/z* 295.1810); ADB: 19-(*Z*)-anhydrovobasinediol (*m/z* 309.1881); HDK: 3-hydroxykoumidine (*m/z* 311.1760); G-N: gelsemine N-oxide (*m/z* 339.1709); MADB: Na-methoxy-19(*Z*)anhydrovobasinediol (*m/z* 339.2071); VAP: 19*E*-16-*epi*-voacarpine (*m/z* 369.1814); G-A: gelsempervine A (*m/z* 383.1978). **(D)** Koumine-type (KMN-type) alkaloids. KMN: kouminol (*m/z* 325.1916). **(E)** Yohimbane-type (YHB-type) alkaloids. SPV: sempervirine (*m/z* 273.1370). **(F)** Gelsedine-type (GSD-type) alkaloids. MGD: Nb-methylgelsedilam (*m/z* 329.1271); 14-HDG: 14-hydroxygelsenicine (*m/z* 343.1658); DHGM: 4,20-dehydrogelsemicine (*m/z* 357.1814); DHGN: 11,14-dihydroxygelsenicine (*m/z* 359.1607); GSM: gelsemicine (*m/z* 359.1951); 11-HDG:11-hydroxygelsemicine (*m/z* 373.1763); HG: hydroxylation of gelsemicine (*m/z* 375.1924); ATG: 14-acetoxygelsenicine (*m/z* 385.1763); MDHG: 11-methoxydihydrogelesemine (*m/z* 389.2085); AHDG: 14-acetoxy-15-hydroxygelsenicine (*m/z* 401.1713); MHDG: 11-methoxy-19-hydroxygelselegine (*m/z* 405.2009); GSZD: gelseoxazolidinine (*m/z* 429.2030).

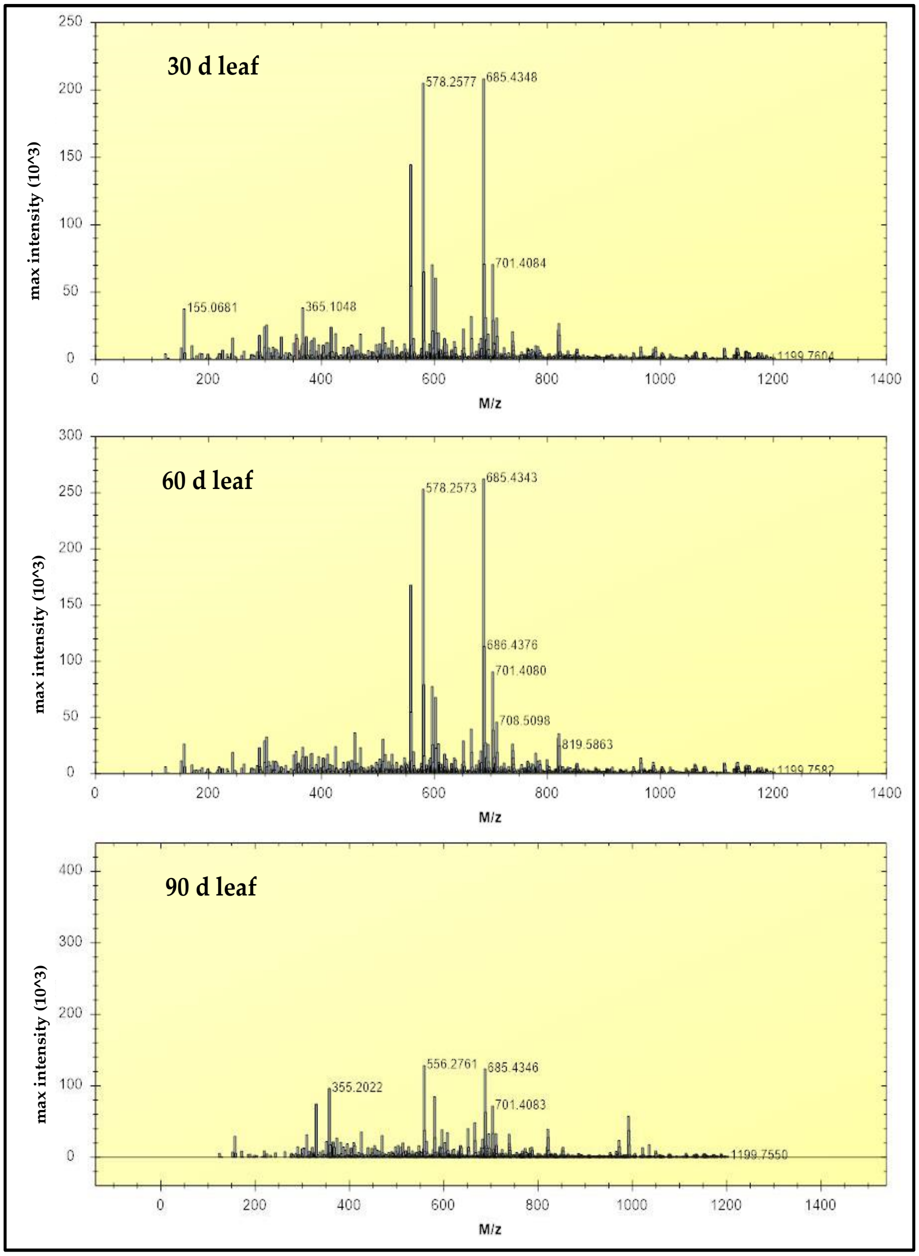

Figure S11. The DESI-MSI spectrums of seedling leaves.

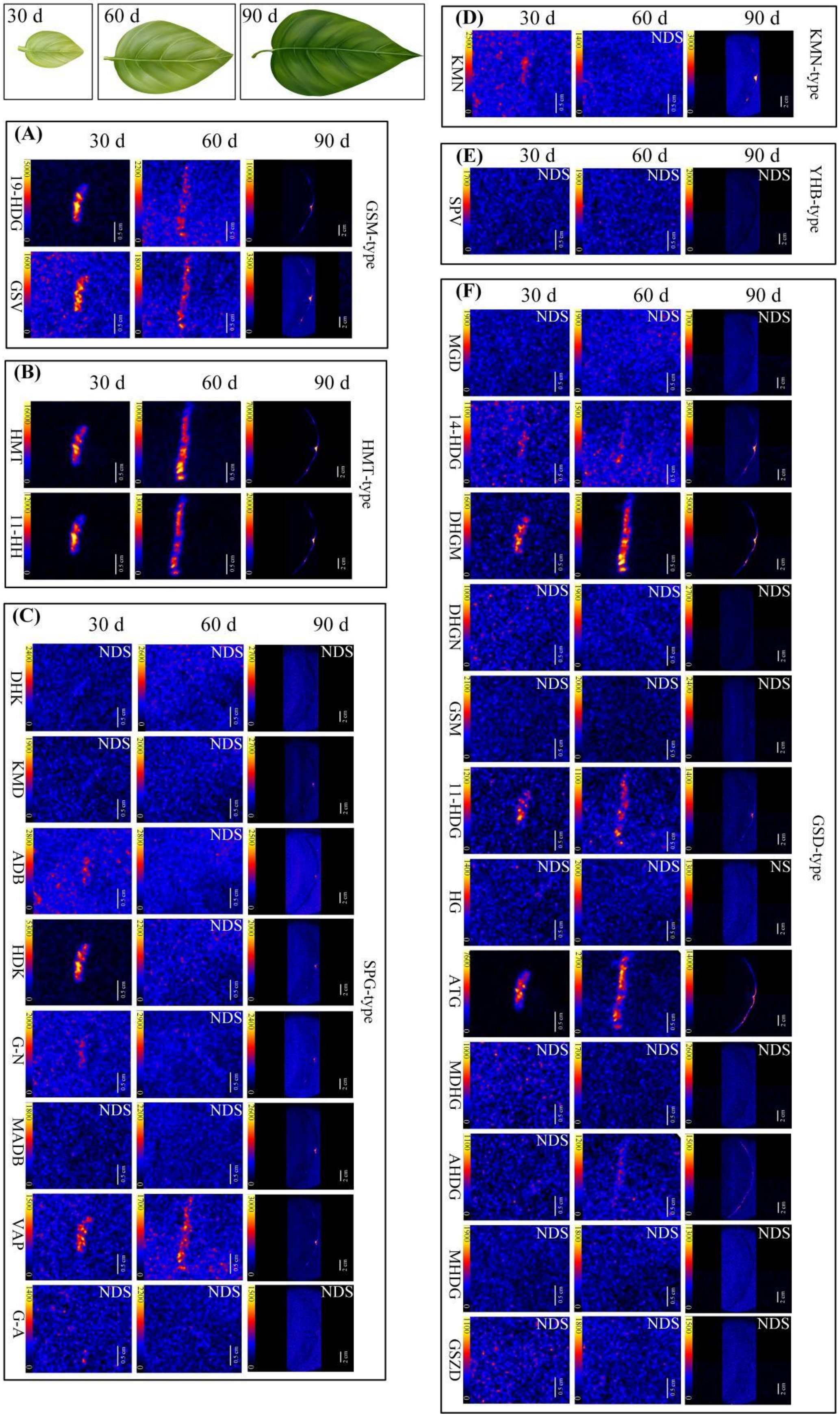
30 d
60 d
90 d
(A)
19-HDG
GSV
GSM-type
(B)
HMT
11-HH
HMT-type
(C)
DHK
KMD
ADB
HDK
G-N
MADB
VAP
G-A
SPG-type
(D)
KMN
KMN-type
(E)
SPV
YHB-type
(F)
MGD
14-HDG
DHGM
DHGN
GSM
11-HDG
HG
ATG
MDHG
AHDG
MHDG
GSZD
GSD-type
NDS
NS

Figure S12. *In situ* visualization of alkaloids in seedling leaves. The sizes of the pattern photos of seedling leaves correspond to the sizes of the DESI-images. NDS means no detectable signal. Each organs/tissues had three replicates and analyzd by DESI-MSI. The names of the alkaloids are abbreviated. **(A)** Gelsmine-type (GSM-type) alkaloids. 19-HDG: 19-(*R*)-hydroxydihydrogelsemine (*m/z* 341.1865); GSV: gelsevirine (*m/z* 353.1865). **(B)** Humantenine-type (HMT-type) alkaloids. HMT: humantenine (*m/z* 355.2022); 11-HH: 11-hydroxyhumantenine (*m/z* 371.1971). **(C)** Sarpagine-type (SPG-type) alkaloids. DHK: dehydrokoumidine (*m/z* 293.1644); KMD: koumidine (*m/z* 295.1810); ADB: 19-(*Z*)-anhydrovobasinediol (*m/z* 309.1881); HDK: 3-hydroxykoumidine (*m/z* 311.1760); G-N: gelsemine N-oxide (*m/z* 339.1709); MADB: Na-methoxy-19(*Z*)anhydrovobasinediol (*m/z* 339.2071); VAP: 19*E*-16-*epi*-voacarpine (*m/z* 369.1814); G-A: gelsempervine A (*m/z* 383.1978). **(D)** Koumine-type (KMN-type) alkaloids. KMN: kouminol (*m/z* 325.1916). **(E)** Yohimbane-type (YHB-type) alkaloids. SPV: sempervirine (*m/z* 273.1370). **(F)** Gelsedine-type (GSD-type) alkaloids. MGD: Nb-methylgelsedilam (*m/z* 329.1271); 14-HDG: 14-hydroxygelsenicine (*m/z* 343.1658); DHGM: 4,20-dehydrogelsemicine (*m/z* 357.1814); DHGN: 11,14-dihydroxygelsenicine (*m/z* 359.1607); GSM: gelsemicine (*m/z* 359.1951); 11-HDG:11-hydroxygelsemicine (*m/z* 373.1763); HG: hydroxylation of gelsemicine (*m/z* 375.1924); ATG: 14-acetoxygelsenicine (*m/z* 385.1763); MDHG: 11-methoxydihydrogelesemine (*m/z* 389.2085); AHDG: 14-acetoxy-15-hydroxygelsenicine (*m/z* 401.1713); MHDG: 11-methoxy-19-hydroxygelselegine (*m/z* 405.2009); GSZD: gelseoxazolidinine (*m/z* 429.2030).
